# Insights into genetic aberrations and signalling pathway interactions in chronic lymphocytic leukemia: from pathogenesis to treatment strategies

**DOI:** 10.1186/s40364-024-00710-w

**Published:** 2024-12-28

**Authors:** Anna Sobczyńska-Konefał, Monika Jasek, Lidia Karabon, Emilia Jaskuła

**Affiliations:** 1 L. Hirszfeld Institute of Immunology and Experimental Therapy, Polish Academy of Sciences, Rudolf Weigl 12, 53-114 Wroclaw, Poland; 2 Lower Silesian Oncology Hematology and Pulmonology Center, Ludwik Hirszfeld square 12, 53-413 Wroclaw, Poland

**Keywords:** CLL, BTK, BCR, TP53, HSM, IgHV, ROR1

## Abstract

Chronic lymphocytic leukemia (CLL) is prevalent in adults and is characterized by the accumulation of mature B cells in the blood, bone marrow, lymph nodes, and spleens. Recent progress in therapy and the introduction of targeted treatments [inhibitors of Bruton's tyrosine kinase (BTKi) or inhibitor of anti-apoptotic B-cell lymphoma-2 (Bcl-2i) protein (venetoclax)] in place of chemoimmunotherapy have significantly improved the outcomes of patients with CLL. These advancements have shifted the importance of traditional predictive markers, leading to a greater focus on resistance genes and reducing the significance of mutations, such as *TP53* and del(17p). Despite the significant progress in CLL treatment, some patients still experience disease relapse. This is due to the substantial heterogeneity of CLL as well as the interconnected genetic resistance mechanisms and pathway adaptive resistance mechanisms to targeted therapies in CLL. Although the knowledge of the pathomechanism of CLL has expanded significantly in recent years, the precise origins of CLL and the interplay between various genetic factors remain incompletely understood, necessitating further research. This review enhances the molecular understanding of CLL by describing how BCR signalling, NF-κB PI3K/AKT, and ROR1 pathways sustain CLL cell survival, proliferation, and resistance to apoptosis. It also presents genetic and pathway-adaptive resistance mechanisms in CLL. Identifying B-cell receptor (BCR) signalling as a pivotal driver of CLL progression, the findings advocate personalized treatment strategies based on molecular profiling, emphasizing the need for further research to unravel the complex interplay between BCR signalling and its associated pathways to improve patient outcomes.

## Background

Chronic lymphocytic leukemia (CLL) is the most commonly diagnosed type of leukemia among adults in the Western world, accounting for 25–30% of all leukemias. CLL is characterized by clonal proliferation and accumulation of mature B cells in the blood, bone marrow, lymph nodes, and spleen [1]. The International Workshop on Chronic Lymphocytic Leukemia (iwCLL) provides guidance for establishing the diagnosis of CLL, which requires the evaluation of blood counts, blood smears, and immunophenotypes. The diagnosis of CLL requires the presence of at least 5000 B-lymphocytes per microliter of peripheral blood for a minimum duration of three months. It is also necessary to confirm the clonality of circulating B-lymphocytes using flow cytometry. The leukemia cells identified in the blood smear were small mature lymphocytes with a narrow cytoplasmic border and dense nuclei lacking discernible nucleoli. Additional morphological features commonly associated with CLL are Gumprecht nuclear shadows or smudge cells, which are found as cellular debris. Recent studies have shown that diagnostic panels, including CD19, CD5, CD20, CD23, kappa, and lambda, are usually sufficient to confirm the diagnosis [2].

**Fig. 1:**
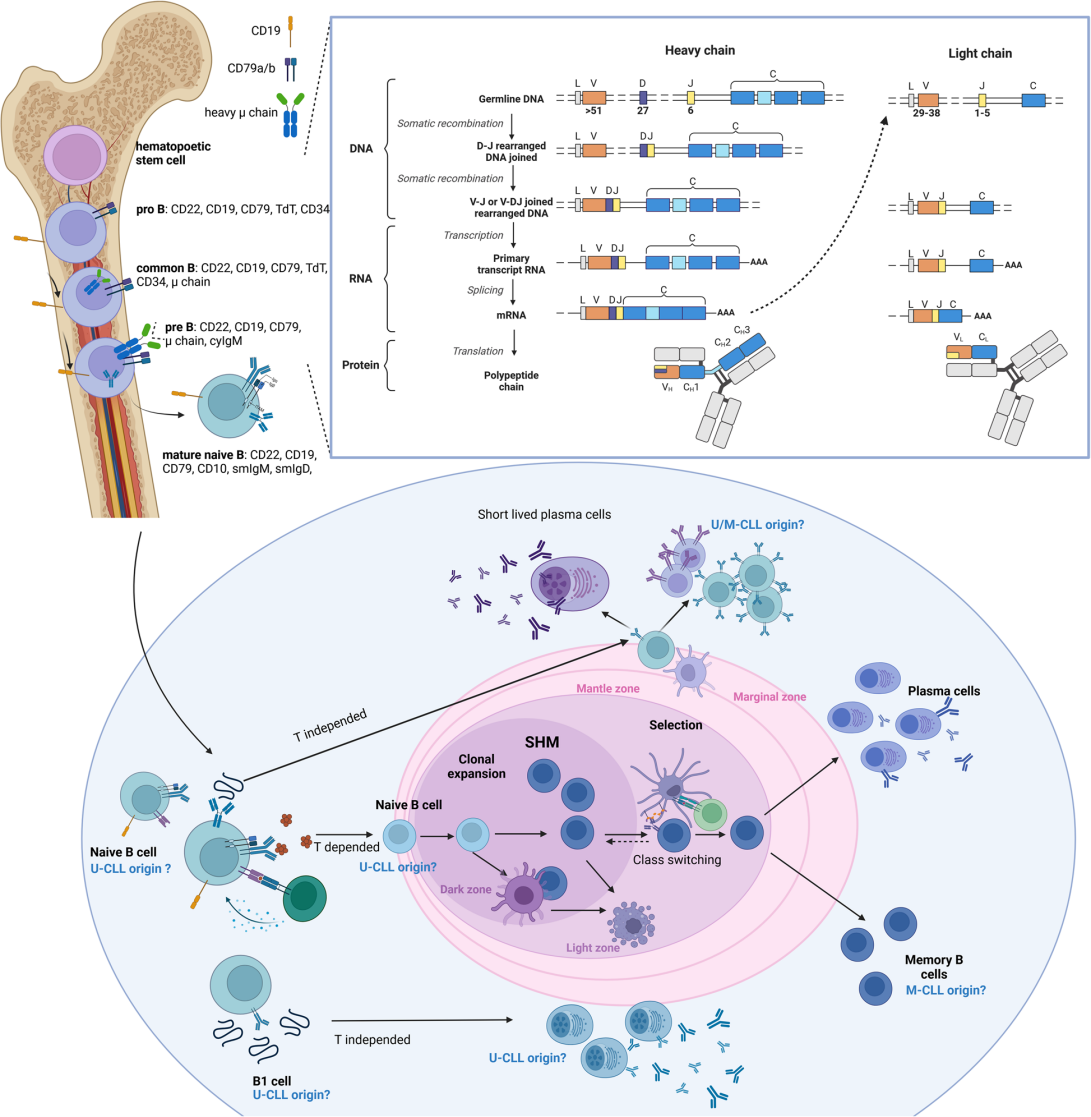
The complex differentiation process of B cells and key B cells stages where disruptions can lead to the development of CLL. During B lymphocyte development, the recombination of VDJ segments in the immunoglobulin heavy chain and VJ segments in the light chain occurs, leading to a diverse repertoire of B cell receptors (BCRs) and immunoglobulins. This process, known as V(D)J recombination, proceeds as follows: the germline sequence of immunoglobulin chains consists of V, D, and J segments. During B lymphocyte maturation, the recombination-activating genes (RAG1 and RAG2) initiate the precise cutting and rejoining of first DJ segments and then VDJ segments for the heavy chain, and VJ segments for the light chain (which lacks D segments). This results in millions of possible segment combinations, contributing to the diversity of BCRs. VDJ recombination starts in the pro-B lymphocyte with the rearrangement of DJ segments on both chromosomes. Subsequently, the V segments rearrange with the previously formed DJ segments on one chromosome. A surrogate light chain is then attached to the newly formed heavy chain, and a check is performed to ensure the antibody is productive and capable of recognizing an antigen. If the first heavy chain is non-functional, the process shifts to the second chromosome. Upon obtaining a productive heavy chain, the light chain rearrangement begins with the kappa chain. If this antibody does not bind the antigen properly, rearrangement occurs on the second kappa chromosome. If unsuccessful, the process moves to the lambda chain. This continues until a functional BCR that binds the antigen with appropriate affinity is produced. The B lymphocyte then migrates to the proliferation center of the lymph node, where clonal expansion and somatic hypermutation (SHM) occur. In the light zone of the lymph node, follicular dendritic cells (FDC) and follicular helper T cells (TFH) facilitate the selection of the best-matched receptors. Centrocytes, in a state of activated apoptosis, compete for survival signals from FDCs and TFH cells. B lymphocytes can return to the dark zone for additional SHM. They then exit the lymph node, differentiating into effector B lymphocytes (which undergo apoptosis after antigen elimination), long-lived plasma cells, or memory cells. B cells that undergo T-independent activation migrate to the marginal zone, proliferate, and leave the node as short-lived plasma cells or effector cells. These cells characteristically produce natural antibodies, which are polyreactive. The figure shows a potential origin of chronic lymphocytic leukaemia (CLL), considering the presence or absence of SHM, indicating mutated (M-) and unmutated (U-) CLL. Adapted from “B Cell Receptors (Light and Heavy Chains)”, created by Akiko Iwasaki and Jung-Hee Lee using BioRender.com (2012). Retrieved from https://app.biorender.com/biorender-templates. Inspiration from [3]

CLL is believed to originate from B cells that are exposed to antigens and undergo transformation. However, the precise mechanism of this process is still under investigation. Several theories suggest that CLL may originate from the marginal zone, transitional, or human B-1-like B cells [3]. One potential origin is memory-like B cells that exhibit signs of germinal center passage with hypermutated *IGHV* genes. Transitional B cells, which have autoreactive B-cell receptors (BCRs) and both mutated and unmutated *IGHV*, are also considered potential origins of CLL. These cells are at a critical point where changes can direct them through different developmental pathways influenced by BCR interactions, with or without T cell help, leading to class switching. The third type of cells explored as a potential source of CLL is human B-1-like cells, known for their unmutated *IGHV* genes and ability to respond quickly to microbial and apoptotic antigens. However, the connection between B1 cells and the development of mutated *IGHV* CLL is not clear, and the exact human counterpart of B1 cells has yet to be confirmed [4, 5] (Fig. 1).

Patients with CLL have highly heterogeneous clinical courses [6]. Some patients survive for more than ten years without requiring treatment, whereas others experience rapid disease progression and poor outcomes [7]. Years of research to understand the biological mechanisms involved in CLL development and progression have made it possible to identify the diagnostic and predictive factors. Such an approach, in turn, allows for the selection of the most appropriate treatment based on genetic data before implementing therapy [8].

Molecular and cytogenetic diagnostics are crucial for accurate prognosis and treatment choice in CLL. Structural chromosomal abnormalities are common in CLL and are found in > 80% of CLL cases [9]. Over the years, fluorescence in situ hybridization (FISH) analysis has become the gold standard for cytogenetic risk stratification in CLL. Chromosomal abnormalities frequently found in CLL include del(13q), trisomy 12, del(11q) [*ATM* gene], and del(17p) [*TP53* gene]. Other abnormalities have also been identified, such as gains in chromosome 2p or 8q, and deletions in chromosome 6q. The iwCLL recommends using interphase FISH in clinical practice before every new line of treatment, as well as in clinical trials [10]. In addition, karyotype determination and complex karyotypes are becoming increasingly important, particularly for relapses in CLL and Richter transformations (RT) [11].

The use of next-generation sequencing (NGS) has provided deep insights into the mechanisms of CLL pathogenesis, helping to identify small subclones of CLL cells (even as low as 1%) and characterize the genomic landscape of this disease through whole-genome sequencing (WGS) and/or whole-exome sequencing (WES) [12, 13]. The application of WGS and WES has enabled the identification of 44 recurrently mutated genes and 11 recurrent somatic copy number variations (CNV) related to CLL pathogenesis, according to Landau et al. (2015) [14]. These include among others the following genes: *NOTCH1*, *MYD88*, *TP53*, *ATM*, *SF3B1*, *BIRC3*, *FBXW7*, *POT1*, *CHD2*, *RPS15*, *IKZF3*, *ZNF292*, *ZMYM3*, *ARID1A*, and *PTPN11* [15, 16]. Several clinical studies have demonstrated the prognostic impact of these alterations on the time to first treatment (TTFT), progression-free survival (PFS), and overall survival (OS). Notably, *NOTCH1*, *SF3B1*, *BIRC3*, and *TP53* mutations have been associated with unmutated *IGHV* and an unfavourable prognosis [17]. Genetic alterations in the CLL cluster in the characteristic pathways included BCR and toll-like receptor (TLR) signalling (*BTK, IGHV, PLCγ2, EGR2, BCOR, MYD88, TLR2, IKZF3*), DNA damage response (*ATM, TP53, POT1*), NOTCH1 signalling (*NOTCH1* and *FBXW7*), apoptosis (miR15/16 and *BCL2*), NF-κB signalling (*BIRC3, NFKBIE, TRAF2, TRAF3*), and RNA splicing and metabolism (S*F3B1, U1, XPO1, DDX3X*, and *RPS15*).

The results of recent studies utilizing WES and WGS on large datasets have significantly advanced our understanding of the biological underpinnings of CLL and its molecular subtypes. In particular, the study conducted by Robbe et al. (2022) [18] provides a comprehensive map of structural alterations and global features of CLL, including telomere length, mutational signatures, and genomic complexity. Based on their findings, the authors defined five genomic subgroups (GSs) of CLL that correlate with PFS and OS. These subgroups are categorized based on the *IGHV* mutational status:

1. u-GS1 (Unmutated *IGHV* Group 1): Characterized by high-risk features such as *TP53* disruption, short telomeres, and mutations in pathways like MAPK and PI3K. This group lacks a DNA damage response signature and is associated with poor clinical outcomes.
2. u-GS2: Defined by alterations in *ATM*, *BIRC3*, and deletions at 11q22.2–22.3, along with mutations in DNA damage response pathways. Patients in this subgroup are predominantly male and do not exhibit *TP53* mutations or significant genomic complexity.
3. u-GS3: Exhibits a high number of mutations in known and putative coding drivers, introns, and UTRs, along with copy number gains such as trisomy 12 and *NOTCH1* mutations. This subgroup is enriched for older patients and is associated with aggressive disease.
4. m-GS1 (Mutated *IGHV* Group 1): Similar in genomic features to u-GS1 and u-GS2, this group is enriched for older male patients and BCR IG subset 2. Most patients do not exhibit any defined CLL stereotype.
5. m-GS2: Characterized by a high mutation burden in enhancers, UTRs, and promoters, and enriched for del(13q4.2). This subgroup shows a favorable PFS and suggests a potential cure after chemoimmunotherapy.

In a subsequent study by Knisbacher et al. (2022) [19], genomic, transcriptomic, and epigenomic data from 1,148 patients were analyzed, leading to the identification of 109 new candidate genetic drivers of CLL. Although many of these newly discovered genes were mutated at low frequencies, nearly a quarter of the patients harbored at least one mutation in a new putative driver gene. Among these genes were *MAP2K2, DIS3, DICER1, INO80, RFX7, ARSI, TCOF1, CD74, RPS14, UCP2*, and *UCP3*. This study also revealed that *IGHV* subtypes are enriched in unique genetic alterations, leading to divergent clonal trajectories. Specifically, unmutated *IGHV* CLL (U-CLL) was characterized by a significant increase in genetic heterogeneity compared to mutated *IGHV* CLL (M-CLL). Furthermore, based on RNA sequencing (RNA-seq) data from 603 treatment-naive CLL samples, the authors identified eight expression clusters (ECs) that were strongly associated with *IGHV* mutational status and/or epigenetic subtypes “epitypes” that were defined by Kulis et al. in 2012 [20]. Multivariable analyses, which included clinical features and *IGHV* status, confirmed the independent prognostic impact of these ECs on both TTFT and OS. This underscores the importance of integrating genomic and transcriptomic data to enhance prognostic stratification and therapeutic decision-making in CLL.

The discovery of these ECs expands the contemporary disease framework, as specific ECs were associated with *IGHV* status, epitypes, and genetic events. Yet, none of these previously defined groups completely captured the phenotypic diversity exhibited in the expression profiles. Additionally, identifying discordant cases with gene expression profiles inconsistent with their *IGHV* status was prognostic, with alterations in *CHD2* potentially contributing to this changed phenotype in M-CLL. By integrating these biological insights with patient outcomes, the study highlighted the prognostic implications of even rare genetic events within *IGHV* subtypes, such as mutations in *ZC3H18* and *RFX7*. Incorporating these data into a unified model revealed the importance of integrating multiple data layers in this disease. Critical components associated with outcomes included the ECs, new genetic alterations such as loss of 5q32, and known factors including the cell of origin (*IGHV* status and epitype), proliferative history of the cell and mitotic clock score called epigenetically-determined Cumulative MIToses (epiCMIT), 17p deletion, and *SF3B1* mutations [19].

Recent advancements in therapy and the introduction of targeted treatments have significantly improved outcomes for CLL patients. Traditional immunotherapies, including monoclonal antibodies, continue to play a crucial role in the CLL therapeutic landscape, even with the widespread use of BTK and BCL2 inhibitors. Monoclonal antibodies, such as rituximab and obinutuzumab, are often used in combination with new inhibitors, exemplified by the regimen of venetoclax plus obinutuzumab, which has shown promising results in CLL treatment [21]. The emergence of new therapies, such as CAR-T cell therapy, has the potential to further revolutionize treatment approaches, and it will be interesting to see how CAR-T therapy impacts patient outcomes in the future. Notably, the U.S. Food and Drug Administration (FDA) approved the CAR T-cell therapy lisocabtagene maraleucel on March 14, 2024, for the treatment of CLL/SLL that has not responded to previous treatments [22, 23].

This evolving treatment landscape has altered the relevance of predictive markers such as *TP53* mutations and del(17p), yet disease relapse remains a significant challenge due to the substantial heterogeneity of CLL and the complex interplay of genetic resistance mechanisms and pathway-adaptive resistance mechanisms. This review aims to enhance the molecular understanding of CLL by exploring the critical role of BCR signalling and its associated pathways, such as NF-κB, PI3K/AKT, and ROR1, in sustaining CLL cell survival, proliferation, and resistance to apoptosis. We describe the genetic resistance mechanisms and adaptive resistance pathways that complicate treatment, emphasizing the need for a comprehensive approach to address these challenges. Furthermore, we present the current understanding of how the genetic landscape influences the intricate networking that exists both within and between signalling pathways linked to the development of CLL. These insights may guide future research and clinical practice aimed at improving patient outcomes.

### B cell receptor (BCR)

One of the most essential features of B cells is their ability to synthesize a virtually infinite repertoire of antibodies [24] that can recognize and bind to nearly every antigen [25]. Antibodies produced by B cells are either secreted or membrane-bound. Membrane-bound antibodies are B-cell receptors [25]. It is estimated that the diversity of the native antibody repertoire accounts for approximately 10^13^ unique sequences [25, 26]. Such a diverse repertoire of antibodies may be created due to the way how they are encoded. Antibodies are composed of heavy and light chains encoded by many gene segments that are spliced together in the process of random recombination of different variable (V) gene segments with diversity (D) and joining (J) gene segments (V(D)J recombination). The light-chain variable region (Fv) is encoded by the variable segment (V) and joining segment (J). The heavy chain is encoded apart from the variable and joining segments, in addition by diversity (D) segments [25]. The high variability in BCRs partially stems from the large number of V, D, and J gene segments in both immunoglobulin (IG) chains. For the heavy chain, sources indicated more than 51 V, 27 D, and 6 J genes [27, 28]. Apart from junctional diversity related to rearrangements, an additional level of diversification is caused by the occurrence of random insertions and deletions of non-templated (N) nucleotides at the IGHD-IGHJ, IGHV-IGHD and IGLV/IGKV-IGLJ/IGKJ junctions. Similarly, functional IG light-chain genes, either of the IG kappa (IGK) or lambda (IGL) variant, are created by combining 1 of approximately 40 IGK variable (IGKV) genes with 1 of approximately 5 IGK joining (IGKJ) genes, or 1 of approximately 30 IGL variable (IGLV) genes with 1 of approximately 4 IGL joining (IGLJ) genes. These mechanisms occur in early-stage B cells (Fig. 1) [24].

The next mechanism that generates additional diversity in BCR is somatic hypermutation (SHM), which is related to the creation of additional mutations due to Activation-Induced Cytidine Deaminase (AID) activity [24]. SHM occurs in the variable domains of immunoglobulin heavy chains (VH) and light chains (VL) [29]. The AID protein converts cytosine (C) to uracil (U), leading to transition/transversion mutations in variable regions of IG [30]. Mutations are generated by replication over U:G mismatches [30], causing a T:A base change and uracil processing by base-excision repair (BER) and mismatch repair (MMR) enzymes [29]. All these events led to introducing a broad spectrum of point mutations characteristic of SHM at a rate significantly exceeding the background mutational level. SHM occurs in B cells, in which the BCR comes into contact with an antigen (Fig. 1) [24, 29].

Patients with CLL are classified into two categories based on the presence and load of accumulated somatic hypermutations within *IGHV* genes [8, 31]. Patients with ≥ 98% *IGHV* sequence identity to their germline counterparts were categorized as unmutated CLL, and those with < 98% identity to germline *IGHV* sequences as the mutated CLL [8, 31]. Immunogenetic studies put forward a hypothesis that M-CLL originates from germinal centre-experienced CD5 + B cells that have undergone SHM. In contrast, U-CLL matures independently of germinal centre (GC) reactions [32, 33].

The *IGHV* mutation status is a powerful and accurate predictor of the clinical course of CLL [8]. Mutated *IGHV* genes are frequently identified in newly diagnosed and asymptomatic patients (apps. 50% [31, 34]). M- *IGHV* is also characterized by the high-affinity binding of BCR [35]. Compared to patients with U-CLL, subjects with M-CLL present a more indolent disease [8]. They display a longer TTFT when managed with “watch and wait” [31] a more durable remission, longer PFS after chemoimmunotherapy (CIT) [31, 35], lower risk of transformation [31], and longer OS [31, 36]. In contrast, U- *IGHV* is often determined among progressive (~ 50–60%) and relapsed/refractory (~ 70–80%) CLL patients [31]. U-CLL patients more frequently have other adverse prognostic genomic aberrations. They are more prone to clonal evolution. BCR signalling is increased with higher proliferation of U-CLL cells [8]. Patients with U-CLL have shorter TTFT, shorter time to the next treatment (TTNT), worse response to chemotherapy/chemoimmunotherapy, are more often resistant to chemotherapy, and have a shorter OS [37].

For the past two decades *IGHV* mutational status constituted the most relevant biological feature to classify CLL and predict the patients' clinical behaviour [38]. A recent decade of intensive research into the characterisation of CLL DNA methylome led to the discovery that DNA methylome contains imprints of the cellular origin of CLL cells and allows for the identification of three epigenetic subtypes “epitypes” of CLL [32, 33]. For the first time, these epitypes were described by Kulis and colleagues in 2012 and were named as follows: native B cell-like (n-CLL), memory B cell-like (m-CLL) and intermediate CLL (i-CLL) [20]. An independent group confirmed this discovery by assessing “the relationship between individual CLLs and variation in DNA methylation programming in normal B cells” [32]. Similarly to the previous study, 3 subtypes of CLL were distinguished, namely low-programmed (LP-CLL), high-programmed (HP-CLL), and intermediate-programmed (IP-CLL). These subtypes correspond to n-CLL, m-CLL and i-CLL, respectively [32, 33]. As far as the putative cell of origin is concerned, it has been suggested that “n-CLLs/LP-CLL could originate from GC-inexperienced memory B cells (MBC), i-CLLs/IP-CLL from GC-experienced B cells that have been early selected to leave the GC and m-CLLs/HP-CLL from GC-experienced B cells that have undergone several maturation-affinity cycles after leaving the GC” [33]. With respect to *IGHV* mutation status, 95% of m-CLLs/HP-CLL correspond to M-CLL and 97% of n-CLLs/LP-CLL correspond to U-CLL [38]. I-CLL/IP-CLL is characterised by an intermediate degree of *IGHV* mutation status 95.9% in relation to n-CLLs/LP-CLL 99.7% and m-CLLs/HP-CLL 92.7% [39].

Several published studies revealed that these three subtypes of CLL of distinctive cellular origin allow for better stratification of patients in terms of clinical prognosis in relation to classical immunogenetic categorization. Patients with n-CLLs/LP-CLL subtype have a poor prognosis and short TTFT; an intermediate prognosis characterises I-CLL/IP-CLL, and m-CLLs/HP-CLL patients have a better prognosis and longer TTFT [32, 38].

The BCR has three complementarity-determining regions (CDRs): CDR1, CDR2, and CDR3 within the heavy-chain variable region and three within the light-chain variable region [40]. The highest diversity in the BCR repertoire arises from CDR3 [41], a key determinant of antigen–binding specificity of the developing BCR [24, 27]. Despite the high diversification of heavy chain complementarity-determining region 3 (HCDR3) in approximately 30–35% of CLL patients, the skewed usage of IGHV in the CLL BCR repertoire has been identified [24, 42]. Moreover, analysis of the amino acid sequences of these BCRs revealed that subgroups of unrelated patients possessed leukemic clones with virtually identical HCDR3s in terms of composition, length, and biochemical properties [24]. This phenomenon has been defined as BCR “stereotypy” and “refers to highly restricted and sometimes identical variable heavy complementarity determining region 3 (VH-CDR3) sequence among different CLL patients” [27] The usage of IGHV in the CLL BCR repertoire is not random since some genes are overrepresented in CLL. These included IGHV1-69, IGHV4-34, and IGHV3-21. In contrast, genes from the IGHV7 subgroup are rarely used by malignant clones [24].

BCR stereotypy is considered the most remarkable feature of the CLL immunogenetic landscape because, despite the typical CLL heterogeneity, stereotyped subsets identified during research are characterized by remarkably consistent biological profiles and clinical pictures [43]. Initially, BCR-stereotyped subsets were established considering the level of VH CDR3 similarity and *IGHV* gene usage. Later, the criteria to be met to determine the BCR stereotype subset were revisited. Regarding VH CDR3, “two independent IGHV-IGHD-IGHJ rearrangements may be assigned to the same stereotyped subset if the respective VH CDR3 has at least 50% identity and 70% similarity at the amino acid sequence level” [43]. It has been shown that 40% of all patients with CLL can be assigned to stereotyped subsets [44]. The series of performed research on a large number of CLL cases allowed for the identification of hundreds of subsets, 19 of which have been classified as “major,” as they represented about 12% of the total investigated CLL subjects [24, 43]. In terms of usage of VH family genes, it has been demonstrated that n-CLLs/LP-CLL predominantly used VH1-69, m-CLLs/HP-CLL most commonly employ VH3-7 and VH4-34, and I-CLL/IP-CLL in majority utilized VH3, including VH3-21 and VH3-23. Of note, it has been demonstrated that IGLV3-21 rearrangements were present in more than 50% of I-CLL/IP-CLL samples [39].

Further investigation demonstrated that patients representing the same stereotyped subset also share, among others, the same pattern of recurrent gene mutations, consistent signatures of gene expression, DNA methylation, antigenic recognition profiles, the similar landscape of antigen reactivity, BCR 3D structure, Toll-like receptor signalling, as well as “classic” and cell-autonomous BCR signalling [43, 45]. More importantly, some of the identified subsets displayed similar clinical features and outcomes, including response to therapy [45]. Subsets #1, #2, #6, and #8 were characterized by a particularly aggressive course of CLL [45], whereas subsets #1, #2, #4, and #8 constituted the largest groups [46]. From a clinical point of view, most attention has been paid to subsets #2 and #8 [45].

CLL subset #2 occurs in approximately 2.5 to 3% of patients, and about 5.5% of cases require treatment, constituting the largest stereotyped subset in CLL [45]. The most characteristic features of this subtype are as follows: 1) usage of IGHV3-21, IGHJ6, and IGLV3-21, with a short 9 – amino acid-long HCDR3; 2) the high frequency of del(13q), del(11q), and *SF3B1* mutations [24]; and 3) poor clinical outcome independent of *IGHV* mutational status [45] since it includes both U-CLL and M-CLL cases [46]. Additionally, subset #2 has been identified in prospective multicenter clinical trials as an independent prognostic marker for shorter TTFT, TTNT, and PFS in CLL, regardless of the *IGHV* mutational status [46]. Even though most cases belonging to this subset have M-CLL, chemo(immune)therapy does not represent an optimal treatment choice for these patients [43].

Subset #8 accounted for approximately 0.5% of all CLL cases [45] and comprised cases with unmutated gene rearrangements of IGHV4-39/IGHD6-13/IGHJ5 paired with IGKV1(D)−39/IGKJ2 gene rearrangements, [46, 47] and displayed the highest risk for Richter transformation among all CLL cases [44, 47]. It is also characterized by a high frequency of trisomy 12 and *NOTCH1* mutations and poor outcomes [43, 44].

It appears that not only the immunoglobulin heavy chain is important for the development of CLL. The significance of the antibody light chain in CLL is substantial, especially in relation to the antigen selection processes. Research has demonstrated that BCR immunoglobulin light chains play a role in these processes, revealing a distinctive biological backdrop for CLL cases expressing IGLV3-21 gene, particularly those with clear signs of somatic hypermutation. This engagement of light chains in antigen recognition suggests a more intricate mechanism for the progression of CLL, where light chains contribute to antigen-driven evolution of the disease. Moreover, limited variable light-chain CDR3 usage in subsets #2 and #169, which employ the IGLV3-21 gene, highlights the potential for antibody light-chain-restricted antigen recognition in CLL, similar to observations in other contexts [44, 47]. This understanding opens new avenues for targeted therapeutic strategies that can specifically disrupt antigen recognition processes mediated by light chains. Recently, it has been shown that all CLL cases of subset #2 carried the IGLV3-21^R110^ mutations [48], which explains why these patients, regardless of the *IGHV* mutational status, have a phenotype and adverse clinical outcomes similar to patients with U-CLL [49]. IGLV3-21^R110^ constitutes SHM-derived G > C mutation, causing glycine (G) to arginine (R) substitution at position 110. It was found that the co - occurrence of R110 and lysine 16 (K16) in one BCR and aspartates (D) at positions 50 and 52 of the YDSD motif in a neighbouring BCR are crucial residues required for homotypic BCR–BCR interaction and triggering autonomous, oncogenic BCR signalling [49, 50]. Until now 2 alleles having K16 and YDSD motifs crucial for enabling effective homotypic BCR-BCR interaction after acquiring R110 as a single-point mutation, namely IGLV3-21*01 and IGLV3-21*04 have been identified [49, 50].

The concept of satellite subsets in CLL is essential for a better understanding of disease pathophysiology as it reveals a more restricted BCR immunoglobulin repertoire. This novel classification scheme highlights the close immunogenetic relationships between major stereotyped subsets and their satellites, suggesting a similar pathophysiology and clonal behaviour among them. These insights have implications for the refined molecular classification of CLL, potentially aiding risk stratification and improving clinical decision-making by acknowledging the nuanced heterogeneity within the disease [44, 47].

CLL heavily depends on BCR signalling for its pathogenesis, including the avoidance of apoptosis, promotion of proliferation, and cell activation. The involvement of BCR signalling in CLL is substantiated by evidence that CLL cells rely on BCR signalling for disease progression. This is further supported by the success of inhibitors targeting BCR-associated kinases, which suggests a crucial role for BCR signalling in the progression of CLL [51, 52]. The following chapters discuss the BCR signalling pathways and resistance mechanisms to targeted therapies in CLL associated with these pathways.

### BCR signalling in normal B and CLL cells

**Fig. 2:**
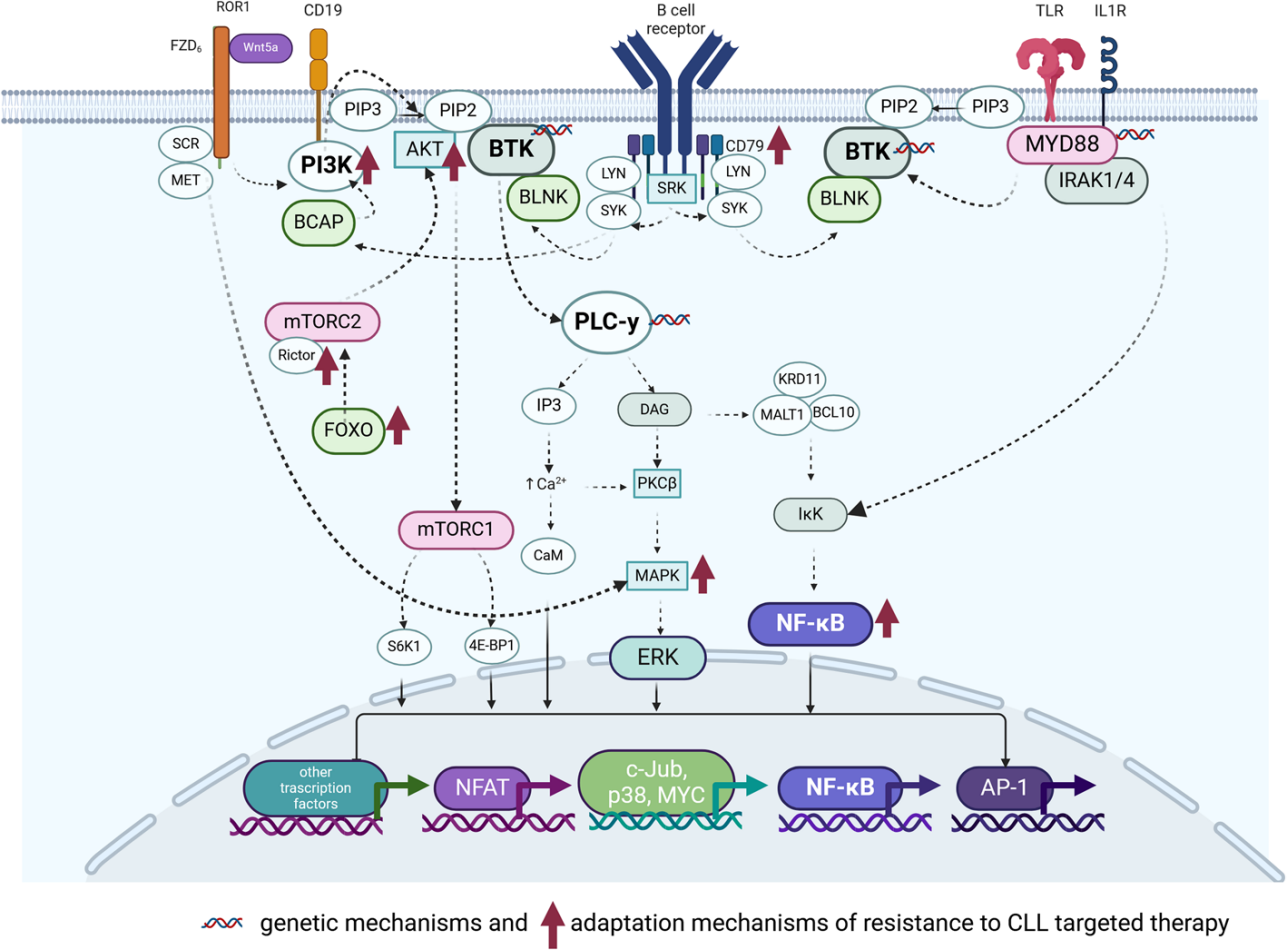
BCR signalling and associated pathways network, genetic resistance mechanisms and pathway adaptive resistance mechanisms to targeted therapies in CLL. BCR signalling activation is initiated when an antigen binds to the receptor, leading to CD79 phosphorylation through LYN and SYK tyrosine kinases. This event forms a signalosome that includes B-cell linker protein (BLNK), Bruton's tyrosine kinase (BTK), and phosphoinositide 3-kinase delta (PI3Kδ). These proteins transduce signals to the calcium-signalling modulator phospholipase Cγ2 (PLCγ2). PLCγ2 catalyses the degradation of phosphatidylinositol-4,5-bisphosphate (PIP2) into inositol 1,4,5-trisphosphate (IP3) and diacylglycerol (DAG), which facilitates the release of calcium from the endoplasmic reticulum (ER). This increase in intracellular calcium promotes the activation of protein kinase C beta (PKCβ), which in turn activates the nuclear factor kappa-light-chain-enhancer of activated B cell (NF-κB) pathway and extracellular signal-regulated kinase (ERK) signalling. The ROR1 pathway, activated by Wnt5a binding, is associated with the BCR pathway and contributes to CLL cell survival and proliferation. The diagram shows the phosphorylation of ROR1 by kinases, such as SRC and MET, leading to downstream activation of PI3K and AKT, which are involved in cell survival and resistance to apoptosis. The TLR signalling pathway involves TLRs recognising PAMPs, leading to recruitment of MyD88 or TRIF adaptor proteins. MyD88 recruits IRAK1/4, forming the Myddosome complex, whereas TRIF interacts with TRAF3 to promote type I interferon production. Both pathways converge on NF-κB activation, which enhances cell survival and inflammatory responses. The figure also indicates the genetic mechanisms of resistance, such as mutations in BTK and PLCγ2, which confer resistance to BCR signalling inhibitors. Adaptation mechanisms have been shown, including alterations in the PI3K pathway and ROR1, which lead to resistance to BCR inhibitors. These mechanisms underscore the dynamic nature of CLL cells in adapting to therapeutic pressure. This visual summary underscores the complexity of signalling pathways in CLL, molecular targets for therapy, and challenges posed by resistance mechanisms. Figure created in BioRender inspiration from [13, 60]

The BCR is attached to the cell membrane via its transmembrane domain. It is tightly and non-covalently connected to a heterodimer consisting of Ig-α (CD79a) and Ig-β (CD79b) molecules. Both CD79a and CD79b contain an immunoreceptor tyrosine-based activation motif (ITAM) in their cytoplasmic tails [53]. The Ig-α/Ig-β (CD79a/CD79b) heterodimer is a signal transduction subunit of the BCR complex [54]. The BCR is linked to a network of kinases and scaffold proteins tethered to the plasma membrane to regulate BCR activation, forming a “signalosome” after antigen binding [55, 56]. Antigen binding initiates membrane movement and aggregation of BCR components, followed by the phosphorylation of CD79a/CD79b ITAMs by the Src-family protein tyrosine kinase LYN and other members of this family, such as Fyn and Blk [52]. These processes allow for the recruitment of spleen tyrosine kinase (SYK) [53, 57] and constitute the first stage of signal transduction from the BCR to the nucleus [54]. LYN and SYK are involved in the phosphorylation of tyrosine residues located in the cytoplasmic tail of B-cell co-receptor CD19, the protein B-cell adaptor for phosphatidylinositol 3-kinase (PI3K) (BCAP), and B-cell linker protein (BLNK), which facilitate the recruitment and activation of PI3K [57]. CD19 and BCAP are involved in PI3K recruitment to the plasma membrane, where PI3K and BLNK participate in Bruton’s tyrosine kinase (BTK) activation, as well as its crucial downstream target, phospholipase Cγ2 (PLCγ2) [54]. BTK activation begins with PH domain-mediated plasma membrane association and transphosphorylation tyrosine at position 551 (Y551) within the BTK catalytic domain. Y551 promoted BTK catalytic activity, resulting in Y223 autophosphorylation of the SH3 domain [58]. Activated BTK phosphorylates PLCγ2, leading to the activation of distal signalling molecules such as inositol 1,4,5-trisphosphate (IP3) and diacylglycerol (DAG). DAG mediates the activation of protein kinase C (PKC)β and subsequent induction of nuclear factor-κB (NF-κB) transcription factor. IP3 releases intracellular calcium (Ca^+2^) and activates the nuclear factor of activated T cells (NFAT). PKCβ is also involved in the activation of mitogen-activated protein kinase (MAPK) family members such as extracellular signal-regulated kinase 1 and 2 (ERK1/ERK2), c-Jun NH2-terminal kinase, and p38 kinase. Recruitment of PI3K to the cell membrane generates phosphatidylinositol-3,4,5-triphosphate (PIP3), which is essential for optimal activation of BTK and protein kinase B (PKB) or AKT recruitment to the membrane [54, 56]. BTK is essential for B cells because, without it, B cells cannot reach functional maturity (Fig. 2) [55, 58]. All these signalling events lead to the third phase of BCR-mediated signalling involving downstream regulators to promote cell proliferation (e.g., Myc), survival (e.g., Bcl-2-interacting mediator of cell death, BIM), and migration through transcriptional modulation and phosphorylation [52, 56, 59].

CD22, CD5, CD72, and FcγRIIB receptors are negative regulators of signalosome signalling, which modulate the duration and intensity of the BCR signal because they possess immunoreceptor tyrosine-based inhibitory motifs (ITIM) in their cytoplasmic tails. During BCR stimulation, LYN phosphorylates these inhibitory motifs. Consequently, the inhibitory lipid SH2 domain-containing phosphatidyl 5-phosphatase 1 (SHIP-1) and protein tyrosine phosphatase (SHP-1) are recruited, and BCR signalling is attenuated [56, 61]. SHIP-1 maintains the dephosphorylation of PIP3 and consequently prevents the recruitment of BTK and PLCγ2 to the cell membrane, thereby reducing the calcium levels. SHP-1 dephosphorylates BTK tyrosine. SHP-1 acts downstream of the CD22 and CD5 receptors, and SHIP-1 acts downstream of FcγRIIB [59]

CLL is considered a BCR-dependent disease because BCR signalling is constitutively activated and constitutes an important biological feature of CLL cells, including CLL cell survival [62]. Two types of BCR signalling have been described, chronically activated BCR and tonic BCR. Chronically activated BCR depend on antigen binding to surface immunoglobulins and employ the canonical nuclear factor-κB (NF-κB) pathway [63]. Tonic BCR signalling does not involve antigen binding, and B cell survival is maintained by PIK3-AKT-mTOR signalling rather than by the NF-κB pathway. Baseline antigen-independent signalling is necessary for the proper development and survival of normal B cells [64]. Tonic BCR activation also occurs in diffuse large B-cell lymphoma (DLBCL) and Burkitt lymphoma. Chronically activated BCR is present in CLL, mantle cell lymphoma (MCL), marginal zone lymphoma (MZL), and activated B-cell (ABC) DBLC. These lymphomas were sensitive to BTK inhibitors [63].

### BCR as a therapeutic target

BCR signalling is pivotal for CLL progression and proliferation, and can be effectively targeted in vivo by inhibiting BTK, a key player in the BCR cascade [65]. Various BTK inhibitors with distinct mechanisms of action are currently in use or under investigation in advanced-stage clinical trials.

BTKi are small molecules that bind covalently or non-covalently near the ATP-binding domain of the BTK protein. By occupying this domain, they prevent the subsequent phosphorylation of BTK, thereby impeding downstream signalling pathways, such as AKT and NF-kB, which regulate cell survival and proliferation. Covalent BTKi include covalent BTK inhibitors (cBTKi) such as ibrutinib, acalabrutinib, and zanubrutinib, whereas non-covalent (ncBTKi) includes pirtobrutinib [65–67]. Ibrutinib was among the first classes to be introduced, and has been widely employed and researched. Nevertheless, it has been associated with cardiovascular comorbidities, including hypertension, arrhythmia, and sudden cardiac death, making it less suitable for patients with preexisting cardiac conditions [35]. Acalabrutinib was developed as a more selective BTK inhibitor, and may possess a superior safety profile compared to ibrutinib. This makes it a potentially safer option for patients with cardiac history or risk [68]. Zanubrutinib, such as acalabrutinib, was designed to be a more selective inhibitor of BTK, potentially providing more durable PFS than ibrutinib, including patients with *TP53* aberrant CLL. Additionally, its safety profile suggests that it may be preferable to ibrutinib in order to minimize certain toxicities [68]. Pirtobrutinib targets ncBTK and can bind to it reversibly, demonstrating efficacy against BTK cysteine 481 (C481)-mutated clones that may emerge during CLL progression following cBTKi treatment. It has shown promising results in patients with relapsed or refractory CLL who have been treated with cBTKi, offering a new treatment option for those who have developed drug resistance [68].

The effectiveness of BTK inhibitors remained consistent across both unmutated and mutated CLL. Clinical trials with the use of ibrutinib in monotherapy, including the RESONATE-2 study or PCYC-1102, reported comparable response rates in U-CLL and M-CLL patients (95% vs. 88 and 87% vs. 81%, respectively) [69]. Similar observations were made for ibrutinib used in combination with rituximab or obinutuzumab (ALLIANCE 041202 study, ECOG-1912 study, iLLU-MINATE study) or with the use of acalabrutinib in the ELEVATE-TN trial [69].

### BTKi treatment resistance

Patients with CLL may acquire resistance to BTKi through various mechanisms. These mechanisms include mutations within the BTK-binding site and in PLCγ2, which is a downstream component of the BCR signalling pathway (Fig. 2). The emergence of different mutations in response to BTK inhibitors is influenced by the specific mechanism of action of each inhibitor, including its binding to the BTK enzyme. Covalent BTK inhibitors, such as ibrutinib, acalabrutinib, and zanubrutinib, form a covalent bond with a C481 residue in the ATP-binding domain of BTK. BTK mutations typically involve substituting Cys481 with another amino acid, disrupting the interaction between the drug and kinase. In contrast, *PLCγ2* alterations are characterized by gain-of-function mutations that enable downstream BCR signalling activation independent of BTK inhibition. The prevalence of mutations, particularly *BTK* mutations, was 3% in first-line patients and increased to 30% in second-line patients who received continuous ibrutinib therapy. Mutations in *PLCγ2* were less frequent in the second-line treatment, reaching a level of 7%, or both genes in 5% of the patients [70]. Both types of mutations, whether in *BTK* or *PLCγ2*, ultimately result in loss of efficacy of BTK inhibitors [63, 65, 71, 72]. BRUIN phase 1–2 clinical trials on pirtobrutinib showed that 70–80% of patients responded to therapy, except those with *PLCγ2* mutations [73]. Moreover, the discovery of "dead-kinase" BTK variants (e.g., Leucine528Tryptophan; L528W) following treatment with zanubrutinib and the subsequent reduction in the efficacy of pirtobrutinib, a non-covalent BTK inhibitor, has generated concern that ncBTKi may not be effective following treatment with selective covalent BTK inhibitors such as zanubrutinib. Furthermore, acquiring non-C481 "dead-kinase" BTK resistance mutations following pirtobrutinib treatment may lead to cross-resistance to second-generation cBTKi [68, 74].

#### Clonal evolution

Within the context of CLL, several types of clonal evolution can be identified, particularly in relation to treatment resistance mechanisms associated BTKi. Understanding clonality in the context of resistance to BTKis is crucial, as it provides insights into how CLL cells adapt and survive under therapeutic pressures.

*Convergent mutational evolution* refers to the acquisition of similar mutations in the same gene across different subclones or patients. This phenomenon has been documented in CLL and it appeared in 26% of patients, affecting nearly 70% of the driver genes associated with the disease [75–77]. For example, mutations in the *BTK* gene, particularly C481S, have been identified in multiple patients as a common mechanism of resistance to BTK inhibitors [78]. The occurrence of these mutations in different patients illustrates the adaptive nature of CLL cells, which can independently acquire similar mutations to survive under selective pressures from therapies.

*Branching Evolution*, on the other hand, is defined by the parallel evolution of multiple competitive clones. This pattern is particularly common during chemoimmunotherapy and ibrutinib therapy, where it is observed in 67% and 25% of patients, respectively [77, 79]. In branching evolution, multiple subclones can emerge and coexist, each potentially harboring different mutations that confer survival advantages under therapeutic pressure. For instance, during ibrutinib treatment, patients may develop various mutations in the *BTK* gene, such as C481S and L528W, which allow different subclones to thrive despite the presence of targeted therapy [76]. This complexity in clonal architecture complicates treatment responses and can lead to treatment resistance.

In minority of patients treated with BTK inhibitors, the emergence of resistant clones has been documented [80]. This type of clonality is *linear evolution* and is characterized by the persistence of a founder clone, which acquires new mutations over time. This type of evolution is particularly prevalent in Richter transformation, where approximately 90% of cases exhibit a linear pattern. In these instances, a single clone evolves into a more aggressive phenotype, often associated with mutations in key genes such as *TP53* [81]. Conversely, linear evolution is less common in CLL progression after chemotherapy, observed in only about 33% of cases and in 7% of patients treated with BTKi [80]. The predominance of linear evolution in RT suggests a more straightforward progression of the disease, where a single clone accumulates mutations that drive its aggressiveness.

In addition to the evolutionary patterns, the growth dynamics of CLL can also be classified into *logistic and exponential growth patterns*. Gruber et al. [82] described that patients exhibiting a logistic growth pattern tend to stabilize their disease without requiring therapy, often harboring favorable genetic features such as deletion 13q14 and mutated *IGHV* status [83]. In contrast, exponential growth patterns are associated with higher genetic complexity and faster disease progression, indicating a more aggressive disease course [84]. This distinction emphasizes the importance of monitoring growth patterns in conjunction with mutational analysis to inform treatment decisions.

The patterns of clonal evolution in CLL—linear evolution, branching evolution, and convergent mutational evolution—reflect the complex interplay of genetic alterations and therapeutic pressures. Linear evolution is predominant in Richter transformation, while branching evolution is common during chemotherapy and ibrutinib therapy. Convergent evolution highlights the adaptive capabilities of CLL cells, allowing them to develop similar mutations across different clones. Understanding these patterns is essential for optimizing treatment strategies and improving patient outcomes in CLL, particularly in the context of BTK inhibitor resistance. The intricate relationship between clonal evolution and resistance mechanisms underscores the need for ongoing research and monitoring to effectively manage this heterogeneous disease.

### Other BTKi resistance mechanisms

The intricacy of the BCR signalling network is increased by simultaneous pathway activation and extensive communication between different downstream signalling molecules. In addition to gene mutations in the signal transduction pathway, resistance to BTK inhibitor therapy may be influenced by adaptive mechanisms originating from various points within the BTK signalling network, as several pathways are involved in signal transduction by BCRs. This is important because it could lead to the development of new mechanisms for resistance to treatment.

One such pathway is the previously mentioned PI3K, a family of enzymes integral to various cellular functions, including growth, proliferation, differentiation, and survival, which often exhibit dysregulation in cancer. These enzymes are categorized into three classes, with Class I PI3Ks comprising of four isoforms. Among these, PI3Kδ is a kinase responsible for amplifying and transmitting signals from the BCR on the cell surface to the downstream AKT signalling pathway, making it a crucial target in CLL [85]. PI3K inhibitors (PI3Ki) are compounds that bind to the ATP-binding pocket of PI3K, blocking a key survival signalling pathway in CLL cells that involves AKT. Furthermore, this pathway can also be targeted by AKT or mTOR inhibitors such as everolimus, which are currently under development [86, 87] (Fig. 2). PI3Kδ isoform has been identified as a potential target in CLL cells [18]. Idelalisib and duvelisib are both selective inhibitors of the delta isoform of PI3K, which have received approval from the FDA in 2014 and 2018, respectively, for the CLL and follicular lymphoma (FL) [88–90]. Their approval was based on pivotal clinical trials that demonstrated significant efficacy in patients with relapsed or refractory disease [88–90]. For instance, idelalisib, in combination with rituximab, has shown improved PFS in patients with CLL [91]. Despite their therapeutic potential, the toxicity profiles of idelalisib and duvelisib have limited their use in clinical practice. Common adverse effects associated with idelalisib include diarrhea, liver function test abnormalities, and an increased risk of infections, which can lead to treatment discontinuation in some patients [88, 92]. Similarly, duvelisib has been associated with significant toxicities, including diarrhea, neutropenia, and immune-mediated adverse events (irAEs) [93–95]. These toxicity profiles necessitate careful patient selection and monitoring, complicating their integration into standard treatment regimens [92, 96]. The dual inhibition of PI3K-δ and PI3K-γ can lead to alterations in T-cell function, contributing to the development of irAEs [92, 94, 95]. For instance, studies have indicated that duvelisib treatment can result in a decrease in naive CD4 + and CD8 + T-cell subsets, along with the accumulation of activated T cells, which may explain the increased incidence of irAEs [95, 96]. This highlights the importance of monitoring immune responses in patients undergoing treatment with these agents, as their immunomodulatory effects can significantly impact overall patient management.

Other BTK networking pathways and adaptive mechanisms are associated with the receptor tyrosine kinase-like orphan receptor 1 (ROR1). The Wnt signalling pathway includes two signalling cascades: β-catenin-dependent and β-catenin-independent, with different functional consequences. The Wnt/PCP pathway constitutes a branch of the β-catenin-independent signalling pathway. The ROR1 coreceptor belongs to this branch. The expression of ROR1 was determined on the surface of CLL cells, but not in mature normal B cells. Additionally, the expression of ROR1 was detected in a subset of the non-malignant intermediate stage B cell precursors in the bone marrow, called hematogones (CD10^+^, CD19^+^, CD45^+^dim, CD34^−^ and TdT), providing a pro-survival signal to these precursors by activating the MEK and ERK pathways [97, 98]. ROR1 is activated by Wnt-5a binding and provides a survival signal for CLL cells. Higher levels of Wnt5a were detected in the plasma of patients with CLL than in healthy subjects [99]. Of note, it has been found that IGLV3-21^R110^ upregulated *WNT5A* [49]. The interaction between ROR1 and Wnt-5a is insufficient for signal transduction because it requires simultaneous binding of Wnt-5a to the Frizzled receptor, which triggers ROR1 phosphorylation at serine and tyrosine residues, and ROR1 homodimerization or heterodimerization with the ROR-2 receptor. ROR1 phosphorylation is mediated by other tyrosine kinases, such as MET, or intracellular kinases, such as LYN and SRC [100]. Next, ROR1 recruits and activates Rho/Rac GTPases, causing enhanced chemokine-directed migration, proliferation, and survival of CLL cells [98, 99]. ROR1 also promotes CLL progression and development [99]. Owing to the importance of survival signals provided by the ROR1 pathway to CLL cells, cirmtuzumab, a humanized IgG1 mAb with a high affinity and specificity for ROR1, was generated to block this signal [99] (Fig. 2). Although over 90% of CLL patients express ROR1, there is variability in ROR1 expression levels among individuals. In 2022, Ghia et al. proposed that ROR1 signalling may upregulate genes associated with drug resistance, suggesting that inhibition of ROR1 signalling could potentially overcome therapy resistance [101]. The Wnt5-a/ROR1 signalling axis was active in patients undergoing treatment with ibrutinib, providing an additional survival signal for CLL insensitive to BTK inhibitors, consequently limiting their capability to eradicate the disease [99]. Considering this, co-treatment with cirmtuzumab (anti-ROR1 humanized monoclonal antibody) and ibrutinib was applied in preclinical studies, which appeared to have a greater effect on the clearance of leukemic cells in a mouse model than monotherapy with either of these agents [99, 100]. The combination of cirmtuzumab and ibrutinib was assessed in a phase I study of 12 patients with CLL (NCT03088878). Therapy was well tolerated and effective with an objective response rate (ORR) of 67% and two complete responses (CR) after 16–48 weeks of treatment. A randomized phase II study comparing cirmtuzumab with ibrutinib and ibrutinib alone is ongoing (NCT03088878) [102].

The creation of antibodies directed against ROR1 has paved the way for the development of antibody–drug conjugates (ADC), bispecific T cell engagers (BiTEs), and chimeric antigen receptor (CAR) T cells [103]. “Given its specificity, in vivo stability, long serum half-life, and potential capacity to concentrate conjugated drugs into lysosomal compartments, e.g. cirmtuzumab appears to be a useful targeting moiety in anti-ROR1 ADCs” [99]. It has been conjugated, *inter alia*, with monomethyl auristatin E (MMAE), a potent antimitotic agent that inhibits cell division by blocking tubulin polymerization [103]. Preclinical studies have confirmed the selective cytotoxicity of this ADC against ROR1-positive CLL cells, which allowed clinical trials to begin with this ADC, known as zilovertamab vedotin (MK-2140, VLS). NVG-111 is a first-class, humanized, tandem scFv, ROR1xCD3 BiTE, which is currently being evaluated in a phase I study of relapsed/refractory CLL and MCL patients who have received at least two prior systematic therapies (ClinicalTrials.gov identifier: NCT04763083) [104, 105]. NVG-111 efficiently killed ROR1^+^ malignant cells. It was designed to engage a unique epitope in the frizzled domain of ROR1. It can redirect T cell activity via a CD3 binder designed to diminish cytokine release [104, 105]. The results obtained for 12 patients who completed a maximum of six cycles of treatment with NVG-111 (median = 3, range 1–6 cycles) provided evidence of NVG-111 antitumor activity as well as promising durability of response to NVG-11, even in CLL patients for whom defective T cell function is frequently observed. The ORR estimated for 11 patients was 55% (6/11). The reported median PFS was 18.7 months [104]. Encouraging data were also obtained for KAN0439834, a tyrosine kinase ROR1 inhibitor that potently induced cytotoxicity against leukemic cells derived from patients with non-progressive or progressive CLL (including fludarabine-resistant CLL cells with and without del(17p)). Moreover, KAN0439834 reduced the number of tumor cells in immunodeficient mice xenotransplanted with human CLL cells [106]. The efficacy of the second-generation ROR1 inhibitor, KAN0441571C, was investigated in leukemic cells derived from the same patients before and after acquiring resistance to ibrutinib. This small-molecule ROR1 inhibitor was equally effective in inducing apoptosis in both ibrutinib-sensitive and -resistant CLL cell lines. Additionally, a synergistic apoptotic effect on ibrutinib-resistant cells was observed for the combination of KAN0441571C and venetoclax [107].

### Apoptosis, regulation of the cell cycle

The most common chromosomal alteration, occurring in approximately 55% of all CLL cases, is del(13q). A benign disease course characterizes the isolated deletion 13q. The size of the 13q deletion was variable; however, the minimal deleted region contained two lncRNA genes (*DLEU2* and *DLEU1*). Moreover, in the critical region of 13q14, miRNA genes (miR-15a and miR-16–1) are located, which regulate the expression of proteins that inhibit apoptosis or are involved in cell cycle progression [1]. These miRNAs also modulate the anti-apoptotic gene, *BCL2*.

**Fig. 3:**
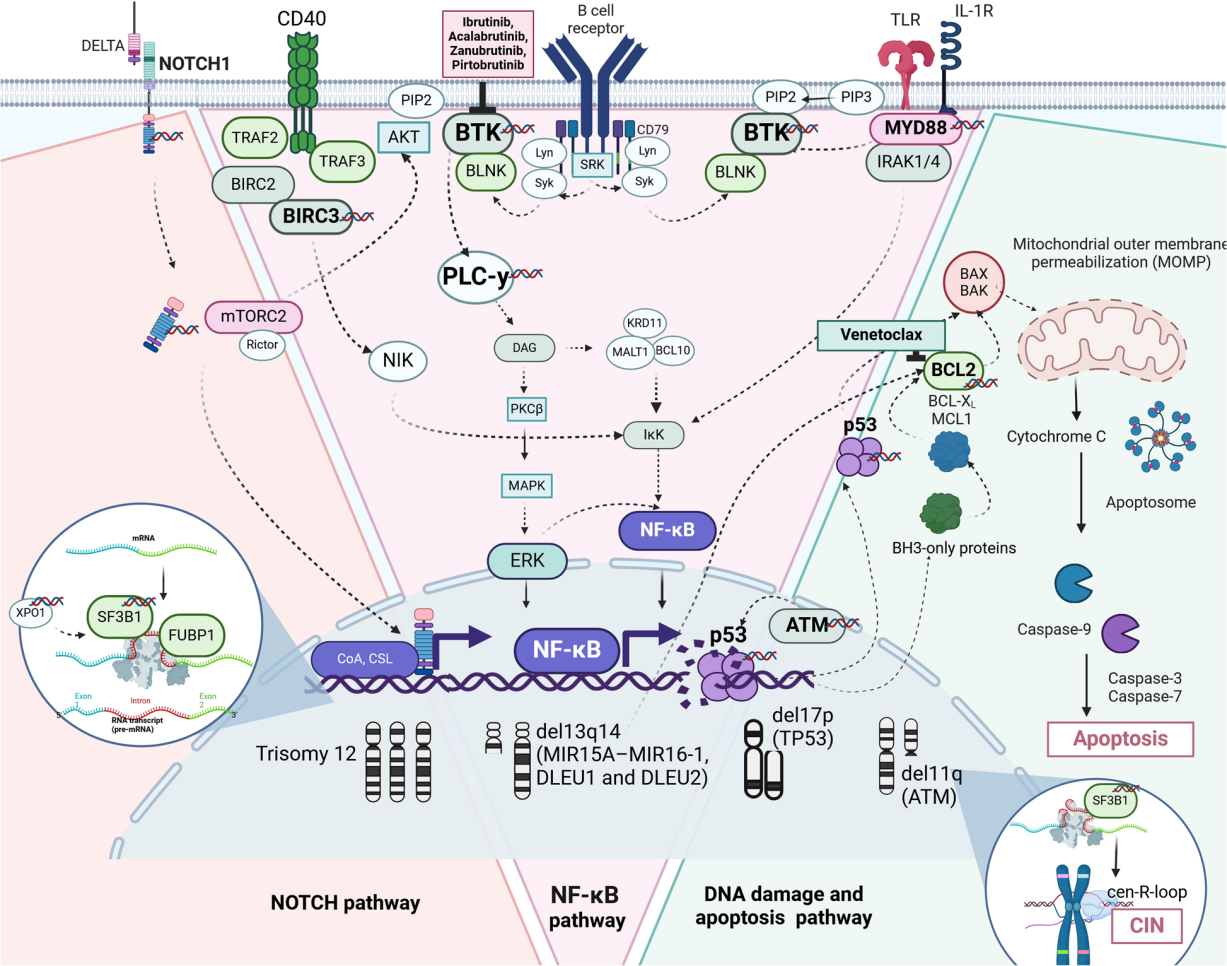
Detailed overview of chronic lymphocytic leukaemia's genetic landscape in the context of signalling network. This figure comprehensively depicts the genetic and molecular aberrations in chronic lymphocytic leukaemia and their involvement in key cellular pathways. The figure illustrates how mutations in specific genes lead to disruptions in several critical cellular processes and signalling pathways: (i) NF-κB Signalling: alterations in *BIRC3* and *MYD88* lead to disruptions in NF-κB signalling, a pathway essential for B cell survival and proliferation. The pathway's components are depicted, including the interactions between various proteins and the downstream effects on transcription factors. (ii) NOTCH1 Pathway: The NOTCH1 signalling pathway is represented, highlighting the consequences of NOTCH1 mutations. The diagram illustrates the interaction between the NOTCH receptor and its ligand Delta, leading to the cleavage and release of the NOTCH intracellular domain (NICD) and the subsequent activation of genes that promote cell survival and proliferation. (iii) RNA Processing: Aberrations in *SF3B1* and *XPO1*, which are involved in RNA splicing and nuclear export, respectively, are shown to affect the processing and function of RNA within the cell. (iv) DNA Damage Response: The figure details the DNA damage response pathway, showing how mutations in *ATM*, *TP53*, impair the cell's ability to respond to and repair DNA damage. It also depicts the connections between these genes and the apoptosis pathway. An element linking RNA processing and the DNA damage response is the *SF3B1* mutation combined with *ATM* deletion, which together drive the accumulation of cen-R-loops. This accumulation disrupts mitotic spindle dynamics and chromosome alignment, thereby cooperating to induce chromosomal instability (CIN). Cen-R-loops interfere with the processes of mitotic spindle formation and chromosome segregation. (v) Apoptosis: The mitochondrial pathway of apoptosis, with a focus on the role of BCL2 and its family members. The binding of venetoclax to BCL2 is emphasised, demonstrating how this interaction leads to mitochondrial outer membrane permeabilisation (MOMP), cytochrome c release, and the subsequent activation of the caspase cascade, ultimately leading to cell death. Figure created in BioRender inspiration from [13, 60, 108].

The BCL2 family includes different proteins, which can be divided into two groups:

1. pro-apoptotic proteins, such as multi-domain (for example, Bcl-2 homologous antagonist/killer—BAK and Bcl-2-associated X protein—BAX) or BH3-only proteins (e.g., Bcl-2-interacting mediator of cell death—BIM and p53 upregulated modulator of apoptosis—PUMA)
2. anti-apoptotic proteins, including BCL2, B-cell lymphoma-extra large—BCL-X_L_, Bcl-2-like protein 2—BCL-W, Myeloid cell leukemia 1 protein—MCL1, Bcl-2-like protein B-BCL-B, and Bcl-2-related protein A1—BFL1 (Fig. 3).

Physiologically, there is a balance between pro-apoptotic and anti-apoptotic factors. Two miRNAs (miR-15a and miR-16–1) bind to specific sequences of *BCL2* mRNA and inhibit BCL2 translation. Deletion of 13q14 (a region that contains miR-15a and miR-16–1) causes a lack of BCL2 inhibition, attenuation of apoptosis, and consequently, promotes CLL cell survival [65].

Considering the significant role of BCL2 in CLL pathogenesis, various compounds targeting this protein have been developed. The first BCL2 inhibitor tested in humans was navitoclax [65, 109]. Unfortunately, it lacks selectivity, as it also targets BCL-X_L_. Subsequent efforts have led to the development of venetoclax, a BH3-mimetic molecule that is now used in several haematological cancers. Venetoclax binds to BCL2, similar to BH3-only proteins, particularly BIM and BID, although with greater affinity. Consequently, it disrupts BCL2's ability to sequester BIM and BID, allowing them to activate the intrinsic apoptotic pathway by interacting with BAX and BAK [110]. Given the promising outcomes of monotherapy and its synergistic potential with other agents, venetoclax has been investigated as a combination therapy in various clinical trials [65].

CLL cells from patients who develop resistance to venetoclax commonly harbor mutations in *BCL2*, particularly mutations resulting in a glycine to valine substitution at the amino acid position 101. Venetoclax demonstrated reduced binding affinity for mutant *BCL2*, thereby diminishing its ability to induce apoptosis in CLL cells [101, 111]. This mutation exhibits similar patterns of convergent evolution, particularly in the context of resistance to venetoclax. The G101V mutation is the most commonly reported alteration associated with venetoclax resistance; however, other mutations such as D103Y and A113G have been identified in patients who relapse on therapy, indicating that multiple mutations can arise to confer resistance [112, 113]. The co-occurrence of these mutations in the same patient further exemplifies the concept of convergent evolution, as different genetic alterations can lead to similar phenotypic outcomes—namely, resistance to treatment [114, 115]. Moreover, recent research has indicated that CLL cells from refractory patients exhibit increased resistance to venetoclax when cultured with accessory cells present in the leukemia microenvironment [116]. However, a critical insight from the research conducted by Jain et al. (2023) in phase II CAPTIVATE study reveals an intriguing aspect of resistance dynamics [117]. While *BCL2* mutations are frequently associated with primary resistance to venetoclax, patients who experience relapse after undergoing time-limited ibrutinib plus venetoclax-based treatments typically do not exhibit these mutations and mutations in *BTK, PLCγ2* genes upon relapse. This observation raises important questions about the underlying mechanisms of resistance, indicating that they may not solely depend on the mutations that have been traditionally associated with treatment failure in CLL. The findings imply that the clonal architecture of CLL is more complex than previously understood. As patients undergo treatment, the selective pressures exerted by therapies like venetoclax may lead to the emergence of alternative resistance mechanisms that do not involve the well-characterized *BCL2* mutations. This suggests that other genetic alterations or adaptive responses within the leukemic microenvironment could play significant roles in the development of resistance. It is plausible that the observed effects result from implementing a time-limited therapeutic strategy, which may play a role in mitigating clonal evolution in CLL [118]. The temporal restriction of therapy could lessen selective pressures that drive resistance-associated clonal expansions, a phenomenon frequently observed in continuous treatment regimens. Moreover, interactions within the microenvironment, including stromal cell support and immune modulation, are known to impact tumor cell behavior and potentially alter treatment outcomes. The mechanisms underlying resistance may not solely depend on the mutations that have been traditionally associated with treatment failure in CLL. Instead, the absence of *BCL2* mutations in relapsed patients points to the possibility of alternative resistance mechanisms that could arise from the complex interactions within the tumor microenvironment. Factors such as Wnt5a have been identified as potential contributors to venetoclax resistance. The activation of ROR1 signalling by Wnt5a leads to the upregulation of ERK1/2 and NF-κB target genes, which may counteract the cytotoxic effects of venetoclax [119, 120]. This highlights the role of the microenvironment in shaping the resistance landscape, suggesting that CLL cells can adapt through mechanisms that do not involve direct mutations in the *BCL2* gene. Moreover, the interplay between *BTK* and *BCL2* mutations in CLL is particularly noteworthy. Studies have shown that the presence of *BTK* mutations can influence the emergence of *BCL2* mutations, indicating a complex relationship where the evolution of one pathway can drive changes in another [121]. For instance, the acquisition of *BCL2* mutations may occur in the context of pre-existing *BTK *mutations, as leukemic cells adapt to the selective pressures imposed by targeted therapies [122]. This interdependence underscores the necessity for comprehensive genomic profiling in CLL to fully understand the spectrum of mutations that contribute to treatment resistance. Further investigation is warranted to delineate the precise mechanisms by which therapy duration and microenvironmental elements converge to affect disease progression and therapeutic response.

### DNA damage response

Deletions in the short arm of chromosome 17 are found in 5–8% of chemotherapy-naïve patients [1]. These deletions always include band 17p13.1, where the *TP53* gene is located, a well-known tumor suppressor that is mutated in various cancers. Patients with del(17p) showed marked resistance to genotoxic chemotherapy. Among the cases with del(17p), the majority (> 80%) had mutations in the remaining *TP53* allele, resulting in *TP53*-biallelic inactivation and poor prognosis. In cases without del(17p), *TP53* mutations are rare, but have a similarly negative effect on chemotherapy response and OS.

*TP53* contains 11 exons that encode p53 protein. There are different forms of *TP53* mutations. First, they may be linked to the loss of 17p by the chromosomal or submicroscopic deletion of LOH (mut/ −). Next, they may occur in the absence of 17p loss in either heterozygous (wt/mut), biallelic (mut1/mut2) or homozygous (mut1/mut1) forms through copy number-neutral loss of heterozygosity (CN-LOH) [7, 123]. The spectrum of *TP53* mutations in human cancers is notable because of their diversity and tissue specificity. In CLL, *TP53* mutations have been detected in almost every codon; however, the majority are within the DNA-binding domain (codons 100–300, exons 4–8). The most common mutations in *TP53*, accounting for approximately 75% of the identified *TP53* mutations, are missense mutations in the *TP53* coding region. This leads to an amino acid change in p53 protein [124, 125]. The expression of mutated p53 protein may result in a lack of activation of the tumor-inhibiting transcriptional response. The pathogenic effect of these mutations is visible, even if they occur in one copy (monoallelic) of *TP53* [126]. In 10–20% of CLL cases, loss of function mutations of *TP53* (missense, nonsense, and frameshift mutations) have been described. *TP53* gene defects tend to be enhanced in cases with unmutated IGH variable regions.

In patients with early-stage asymptomatic CLL, *TP53* abnormalities have been identified in roughly 5- 7% of cases [31]. However, this frequency increases notably to 40% in patients refractory to fludarabine and to 60% in those with Richter syndrome. Disruptions in the *TP53* gene serve as crucial biomarkers in treatment decision-making algorithms for CLL. Specifically, del(17p) and *TP53* mutations consistently correlate with unfavourable disease outcomes in patients undergoing CIT owing to their chemorefractory nature [2, 65]. Therefore, assessing *TP53* aberrations is imperative for patients requiring therapy, with reassessment necessary before initiating subsequent lines of treatment, because of the potential for clonal evolution [127]. In CLL cells with impaired p53 function, DNA damage ailed to induce cell cycle arrest or DNA repair mechanisms, leading to the accumulation of significant DNA alterations. This leads to increased genomic instability, facilitating the emergence of subclones harboring additional genetic mutations that drive CLL progression and transformation [127]. Given the chemorefractoriness associated with *TP53* disruption, *TP53* status is a pivotal determinant for tailoring CLL treatment strategies. This often prompts upfront treatment with BTKi and BCL2i, which can at least partially circumvent the challenges posed by *TP53*-mediated resistance to traditional chemotherapy. Importantly, although BTKi and BCL2i do not directly target the p53 protein, they exert their therapeutic effects through mechanisms that are largely independent of the p53 pathway. This aspect is particularly significant in the current treatment landscape, where direct modulation of p53-related pathways is not the primary focus of therapy. Thus, the reliance on BTKi and BCL2i highlights a strategic shift towards exploiting alternative cellular pathways that can effectively control disease progression in the presence of *TP53* abnormalities [128].

The *TP53* gene exhibits the capacity to encode 12 distinct p53 isoforms through mechanisms including alternative initiation of translation, employment of alternative promoters, and alternative RNA splicing. These isoforms are distinctively expressed in various cancers and possess unique transcriptional activities and tumor-suppressive properties that influence a broad spectrum of biological processes. Isoforms of p53 can perturb this balance with canonical full-length p53 (TAp53), leading to adverse clinical outcomes in specific cancer types. Importantly, ∆133p53 isoforms have been recognized as facilitators of DNA double-strand break repair, thereby protecting cells from death and senescence post-DNA damage. This capacity significantly contributes to the survival of cancer cells, enabling them to evade apoptosis and sustain proliferation, despite genetic insults. ∆133p53 expression is associated with cancer progression, therapeutic response, and patient prognosis in diverse cancer contexts [129–131].

Moreover, independent of genetic mutations, the aberrant expression of p53 isoforms plays a profound role in cancer initiation. Given its pivotal role in cancer biology, including cancer progression and response to chemotherapy, the p53 pathway remains central to oncological research. Clinical investigations have increasingly demonstrated that the specific expression profiles of p53 isoforms correlate with tumorigenesis, treatment efficacy, and prognostic outcomes, with isoform distribution exhibiting variability across different cancer types, underscoring its intricate role in oncogenesis [129]. As the p53 pathway governs a critical network that regulates cell growth and apoptosis, any disruption in the splicing of genes within this pathway could potentially influence the cytotoxic effects of spliceosome inhibitors [132]. Despite the identification of multiple p53 isoforms, their comprehensive biological effects have yet to be fully explored [129].

The second gene associated with DNA damage in CLL is ataxia telangiectasia mutated gene (*ATM*). *ATM* encodes a protein kinase and tumor suppressor that facilitates the repair of DNA double-strand breaks and is an upstream regulator of *TP53*. It also plays a key role in cell division and DNA repair. *ATM* is located in the band q23 on chromosome 11. As with *TP53*, different types of aberrations are observed in *ATM* in patients with CLL. This is predominantly a deletion of the long arm (q) of chromosome 11, which can be found in approximately 10–25% of CLL patients, but somatic mutations in *ATM* are seen in 4–15% of CLL cases. Patients with del(11q) clone typically show massive lymphadenopathy, rapid progression, and shortened OS [1]. Individuals diagnosed with CLL and germline *ATM* variants are diagnosed at a younger age and have a doubled likelihood of experiencing 11q deletion. Furthermore, the specific *ATM* variant p.Leucine2307Phenyloalanine, found in 3% of patients with CLL, correlates with a three-fold higher occurrence of somatic 11q deletion and displays reduced function in cellular assays [133], DNA damage and secondary primary malignancy. It also appears that the cytotoxic effects of chemotherapy, which can damage DNA and lead to aberrations in genes related to DNA repair mechanisms, such as *TP53* and *ATM*, may contribute to the development of secondary malignancies in CLL patients. There seems to be much data discussing the association of secondary malignancies with CLL and their higher incidence in this group of patients. Van der Straten et al. in *Blood Cancer Journal* published the results of their studies of a cohort which included 24 815 CLL patients. In general, patients with CLL face a notably elevated risk of developing secondary primary malignancy (SPM) compared to the average risk observed in the general population [134]. Although no clear genetic evidence has been found, it is important to consider the possibility that CLL patients may be predisposed to developing SPM. The reasons for the increased risk of secondary cancer in patients with CLL remain unclear. In addition to DNA machinery malfunctioning due to mutations, such occurrences could be attributed to individual patient-related risk factors, the presence of a tumor-promoting microenvironment in CLL patients, or chemotherapy-related factors such as immune system suppression. Immunodeficiencies associated with CLL may also make individuals more susceptible to other malignancies [135]. Thus, patients with CLL undergoing treatment with BTK inhibitors may continue to face an elevated risk of SPM because BTK inhibition has potentially deleterious effects on the immune response, and the occurrence of secondary malignancies in CLL and factors contributing to their emergence warrant further investigation. The apparent oversight of this concern may be attributed to the necessity for long-term, comprehensive cohort studies with extensive follow-up periods, complexity of contributing factors, and prioritization of immediate clinical concerns. These challenges have resulted in a paucity of data, leading to several questions regarding the mechanisms underlying SPM development.

### RNA processing

Different genes encoding the spliceosome machinery are mutated in CLL patients. The most frequently mutated gene in this group was splicing factor 3 B subunit 1 (*SF3B1*). It encodes an essential spliceosome component, and mutations probably affect the interaction between SF3B1 and RNA, leading to an altered splicing function [136, 137]. Mutations in *SF3B1* are present in 10–20% of cases [138] and are more frequent in patients with del(11q), del(13q), or trisomy 12. They are usually sub-clonal and expand over time, contributing to disease progression, particularly after chemotherapy. Typically, these missense variants are clustered in the highly conserved HEAT domain of SF3B1. Mutations in *SF3B1* have been implicated in the perturbation of cellular genomic integrity, as evidenced by an increase in DNA damage or an aberrant cellular response to such damage. These mutations result in a partial compromise of the cellular mechanisms responsible for responding to DNA damage, which reflects the dysfunction observed in *ATM* gene mutations rather than that associated with *TP53* gene dysfunction. Notably, cellular samples harboring *SF3B1* mutations, even in the absence of concomitant *ATM* and *TP53* abnormalities, demonstrated partially impaired activation of the ATM/p53-dependent transcriptional and apoptotic pathways in response to DNA-damaging agents. Moreover, *SF3B1* mutations frequently co-occur with deletions at the *ATM* locus on chromosome 11q23, observed in approximately 52–57% of cases, as well as with *ATM* mutations that do not involve 11q deletions [139–141].

The aberrant 3' splice site engendered by *SF3B1* mutations, located 20 bp upstream of the intron, precipitates the truncation of the C-terminal region of the ATM protein. The resultant frameshift mutation led to the production of a truncated protein devoid of the final 720 C-terminal amino acids, which includes critical phosphatidylinositol-3 kinase domains.

To better understand the role of *SF3B1* mutations in CLL, Yin et al. developed a conditional knock-in mouse model with a B cell-specific expression of the *Sf3b1*-K700E mutation, enabling to explore its impact on the disease’s pathogenesis. Their mouse model further investigates whether *Sf3b1* mutation, when combined with *Atm* deletion, leads to increased genomic instability. Moreover, their mouse model showed that co-expression of *Sf3b1*-K700E with *Atm* deletion in B cells leads to the development of low-penetrance CLL, whereas either lesion alone does not [142]. These CLL cells show RNA splicing defects and genomic instability, including recurrent amplifications of chromosomes 15 and 17, suggesting that chromosomal instability (CIN) plays a critical role in CLL initiation [108, 142]. However, the mechanisms through which these mutations contribute to CIN remain unclear.

CIN, a hallmark of cancer, results from errors in chromosome segregation, leading to aneuploidy. Recent studies have highlighted the importance of DNA:RNA hybrids (R-loops) at centromeres (cen-R-loops) in regulating CIN [143].

To investigate whether *SF3B1* mutations contribute to R-loop accumulation and DNA damage in various cellular contexts, Cusan et al. measured R-loop levels in pre-B cell and myeloid cell lines (Nalm-6 and K562) with or without the *SF3B1*-K700E mutation. Their data suggest that *SF3B1* mutations promote unscheduled R-loop accumulation and disrupt the physiological formation and resolution of DNA:RNA hybrids at centromeres. This dysregulation of cen-R-loops likely contributes to CIN [108].

Furthermore, they compared *SF3B1* mutation–associated alternative splice variants with the genomic locations of R-loop peaks. Their findings suggest that altered RNA splicing, driven by *SF3B1* mutations, contributes to R-loop accumulation. Specifically, *SF3B1* mutations generate an alternative splice variant of SERPINE m-RNA-binding protein 1 (*SERBP1*), which encodes a less stable protein with impaired RNA binding, affecting R-loop homeostasis. Together with *ATM* deletion, these mutations cooperate to drive CLL through the accumulation of cen-R-loops [108]. Moreover, *SF3B1*-mutated cells are sensitive to ataxia-telangiectasia mutated and Rad3-related (ATR) kinse and poly (ADP-ribose) polymerase (PARP) inhibitors, which exploit R-loop–induced replication stress, suggesting potential therapeutic synergies with splicing inhibitors and ionizing radiation [108].

Bland et al. in *Nature Genetics* reveal that mutations in *SF3B1* confer selective sensitivity to clinically approved PARP inhibitors (PARPi), independent of homologous recombination deficiency or *BRCA1/2* status [144]. Their findings highlight the potential clinical utility of PARP inhibitors in *SF3B1*MUT cancers, even in the absence of homologous recombination defects. The PiCCLe multicenter phase 1 trial in relapsed leukemia showed that patients with *SF3B1* mutations had the longest progression-free survival when treated with olaparib (PARPi) [145]. Cyclin-dependent kinase 2 interacting protein (CINP) downregulation was confirmed in these patients, further supporting the hypothesis that PARPi may benefit those with *SF3B1* mutations. These results suggest a clinical advantage for PARPi in this subset of patients, and that PARP-trapping agents could offer a new treatment option for patients with *SF3B1*MUT cancers resistant to conventional therapies [144].

Elucidation of the intricate interplay between *SF3B1*, *ATM*, and *TP53* mutations is paramount for identifying novel therapeutic targets and for the stratification of patients with CLL into more refined prognostic categories. This understanding is instrumental to the advancement of personalized therapeutic strategies.

Additionally, lesions in other genes implicated in RNA splicing and metabolism, such as exportin 1 (*XPO1*), have been identified, albeit at lower frequencies. XPO1, a nuclear export protein, regulates the translocation of a plethora of proteins and RNAs from the nucleus to cytoplasm. This protein mediates the nuclear export of several tumor suppressor proteins, including p53, leading to functional inactivation. Overexpression of XPO1 is common in neoplastic cells, attributed to its role in promoting cell survival and proliferation, and is a hallmark of oncogenic phenotypes. In CLL, XPO1 expression is elevated in malignant B cells relative to their normal counterparts, and mutations in *XPO1* have been correlated with high-risk genetic aberrations and accelerated disease progression [146].

Since up to 30% of patients with CLL may have mutations in genes involved in RNA splicing, dysregulation of RNA splicing may be a common mechanism in the pathogenesis of CLL [147].

### Inflammatory pathway and NF-κB signalling

Mutations in the baculoviral IAP repeat containing 3 (*BIRC3*) gene, also called *cIAP2*, impair ubiquitin ligase activity, induce constitutive NF-κB activation, and promote proliferation and survival [148, 149]. This gene is located on the long arm of chromosome 11, proximal to *ATM*. Deletions in 11q include the *BIRC3* locus in approximately 80% of patients with CLL with del(11q) [150]. Moreover, *BIRC3* can be affected by mutations, predominantly nonsense and frameshift variants, with an incidence of 3–5% in untreated patients [151]. Patients with CLL with *BIRC3* mutations are more prone to develop chemorefractoriness, particularly those resistant to fludarabine. The frequency of *BIRC3* mutations increases to approximately 25% in patients refractory to fludarabine [149, 152]. *BIRC3* alterations are associated with high-risk disease, poor outcomes, and PFS and OS [85]. Furthermore, in patients with CLL, an association between *BIRC3* mutations and specific genetic alterations, such as unmutated *IGHV* genes and trisomy 12, has been observed [149]. *BIRC3* mutations lead to continuous activation of NFκB, potentially contributing to treatment resistance and tumor growth by downregulating the p53 protein through MDM2 [85]. The CLL14 phase 3 clinical trial demonstrated shorter PFS in patients treated with chlorambucil-obinutuzumab than in those treated with venetoclax-obinutuzumab, highlighting the significance of *BIRC3* mutations as a biomarker for chemorefractoriness [153]. For these patients, alternative treatments such as cyclin-dependent kinase inhibitors, BTK inhibitors, BCL2 inhibitors, alemtuzumab, and corticosteroids may be considered [154]. Targeting NF-kB and inhibiting this pro-survival pathway represent a potential strategy for treating patients with CLL [155].

Other genes associated with the NF-κB pathway that have been shown to be frequently mutated in CLL include myeloid differentiation primary response 88 (*MYD88*) [156]. MYD88 is an adaptor protein that regulates Toll-like receptor pathways and plays a key role in innate and adaptive immune responses. Following stimulation, MYD88 triggers activation of the NF-kB and MAPK pathways by assembling a signalling complex comprising several intermediary proteins, including IL-1R-associated kinases (IRAKs) and tumor necrosis factor receptor-associated factors (TRAFs), particularly TRAF6 [157]. This mutation initiates the activation of multiple targets, including STAT3 and NF-κB p65 subunits [158] Mutations in this gene have been detected in 2–5% of CLL cases [159]. The most common mutation in *MYD88* is a single nucleotide change (NM_002468.4:c.794 T > C) that leads to the conversion of leucine to proline at position 265 [160]. In CLL, *MYD88* mutations are primarily observed in patients with mutated *IGHV* and deletions of the long arm of chromosome 13 [del(13q)], both of which are associated with a better prognosis and slower disease progression [161]. Multiple studies have shown that patients with *MYD88* mutations have a favourable outcome in CLL, or that there is no association with the course of the disease [162, 163].

Several approaches have been suggested to block the TLR/MYD88 pathway either directly or indirectly. These strategies involve targeting key components, such as IRAK1 and IRAK4 within the myddosome complex, TAK1 in downstream signalling, BTK in the BCR pathway, TLR9 in the My-T-BCR supercomplex, and elements of the concurrently activated PI3K/AKT/mTOR and HCK pathways [157, 164, 165]. Although BTK is not specific to *MYD88* (L265P) and is not directly associated with the MYD88-derived protein complex, BTK inhibition has emerged as a highly effective therapy for CLL. Moreover, MYD88-derived peptides have demonstrated potential to stimulate T-cell responses, suggesting the feasibility of T-cell receptor-based immunotherapy [157, 164]. However, further investigation is needed to fully integrate MYD88-derived treatments into the clinical practice.

### Trisomy 12 and NOTCH1 pathway

Trisomy 12 is observed in 10–20% of patients. It is also associated with intermediate prognosis. The genes involved in the pathogenesis of CLL associated with chromosome 12 trisomy are unknown. However, an association of neurogenic locus notch homolog protein 1 (*NOTCH1*) mutations with + 12 has been observed. *NOTCH1* mutations appear to worsen clinical outcomes in patients with + 12 [166].

The NOTCH family is a highly conserved group of genes that is crucial for regulating hematopoiesis [167]. The NOTCH signalling pathway is involved in many key cellular functions, including proliferation, cell differentiation, and apoptosis. Mutations in *NOTCH1* have been detected in approximately 10% of CLL cases at diagnosis and are associated with unmutated *IGHV* genes and trisomy 12 [168]. Various mutations lead to the deregulation of the intracellular NOTCH1 receptor. In contrast to normal B cells, NOTCH1 is constitutively activated and upregulated in CLL cells and is associated with resistance to apoptosis. CLL patients with *NOTCH1* mutations, compared to those with wild-type *NOTCH1* patients, had a significantly higher probability of developing DLBCL-type RS (45% vs. 4%) [169].

Patients with CLL harboring *NOTCH1* mutations do not benefit from therapies incorporating rituximab, likely due to diminished CD20 expression levels in these patients [138, 170]. Conversely, treatment with alemtuzumab demonstrated prolonged progression-free survival in these patients [171]. Obinutuzumab also exhibits superior efficacy compared to rituximab in patients with CLL with *NOTCH1* mutations [172]. Additionally, exploiting non-coding RNAs as a therapeutic strategy has emerged as a novel approach to target NOTCH signalling [173]. Thus, targeting NOTCH signalling is a promising therapeutic avenue for CLL. Potential mechanisms for targeting NOTCH1 signalling in CLL include utilization of secretase inhibitors (GSIs) and specific anti-NOTCH1 receptor antibodies. The antitumor effects of GSI PF-03084014 were demonstrated in combination with fludarabine in CLL cells with *NOTCH1* mutations [174]. Furthermore, this combination impaired angiogenesis and CXCL12-induced responses associated with tumor migration and invasion [175]. Another approach involves blocking NOTCH ligand-receptor interactions using antibodies. Among the various NOTCH ligands, DLL4 and DLL1 are pivotal in CLL, with DLL4 being the most effective stimulator of NOTCH signalling in cases with *NOTCH1* mutations [176].

## Conclusions and Future Remarks

In this study, we present the key pathways in CLL. Disruption of these pathways in normal B lymphocytes leads to tumor development. However, the inhibition of disrupted pathways could result in the designation of alternative signal communication routes or the selection of clones with mutations resistant to certain drugs. We attempted to present pathway connections and possibilities for alternative communication pathways. It appears that the central axis of CLL pathogenesis is the BCR signalling pathway, which promotes CLL cell survival and proliferation through downstream effectors such as PI3K and NF-κB [177]. The PI3K pathway, activated downstream of BCR, enhances cellular survival and growth and interacts with BCR signalling to modulate cellular responses to external signals [56]. The link between innate immune recognition and B-cell receptor pathways is represented by the TLR pathway, which ultimately promotes survival and proliferation through NF-κB signalling [177, 178]. NOTCH1 signalling in CLL is implicated in cellular fate decisions and interacts with other signalling pathways to influence apoptosis and resistance to cell death, possibly through crosstalk with the PI3K and NF-κB pathways [179]. ROR1 enhances CLL cell survival through Wnt signalling, which can intersect the NOTCH and PI3K pathways, influencing cellular migration and survival [180].

CLL-involved pathways can affect the apoptotic machinery, where disruption leads to the evasion of apoptosis, a hallmark of CLL. The integration of signals from the BCR, PI3K, and NOTCH1 pathways modulates the pro-survival and anti-apoptotic responses [181]. CLL cells often show dysregulation of apoptotic pathways, mainly due to the overexpression of anti-apoptotic proteins such as BCL-2. P53, which influences BCL-2 expression, is also a key player in this process [55]. Moreover, BCR and PI3K pathways can impact apoptosis pathways by promoting the expression of BCL-2 and other related proteins, thereby inhibiting apoptosis and supporting cell survival. Deletion of 13q14, a region that includes BCL2 repressors and microRNAs 15 and 16 which occurs in approximately 50–60% of CLL cases. BCL2 upregulation is driven by hypomethylation and epigenetic dysregulation. This is confirmed by clinical study results, in which venetoclax brings positive therapy outcomes in virtually all patients with CLL, including those without mutations in the *TP53* gene or del(17p) [181].

These pathways share key signalling molecules, such as NF-κB and PI3K, illustrating the complex network of interactions that contribute to the malignant phenotype in CLL, influencing therapeutic strategies [177, 181].

In the scholarly discourse surrounding CLL, *TP53* aberrations encompassing both mutations and deletions [notably del(17p)] have historically been acknowledged as predictors of unfavorable clinical outcomes. These genetic anomalies are associated with a reduced interval to subsequent treatment and diminished or absent therapeutic response to conventional chemo-immunotherapeutic modalities. The adverse effects of these genetic markers were particularly pronounced in a subset of patients with relapsed/refractory (R/R) CLL. Historically, high-risk CLL has been typified by *TP53* alterations and suboptimal response to purine analog-based treatments. Prognostic biomarkers, including unmutated immunoglobulin heavy chain variable region, deletion of chromosome 11q [del(11q)], elevated zeta-chain-associated protein kinase 70 (ZAP70) expression, and increased CD38 expression, correlated with unfavorable prognosis [182]. The importance of efficient DNA repair mechanisms in immunochemotherapy regimens, which can lead to increased DNA damage and an increased need for an effective DNA damage response, is well established. For example, patients with *TP53* aberrations treated with fludarabine, cyclophosphamide, and rituximab (FCR) regimens have shown markedly inferior PFS and OS rates [183].

Recent studies utilizing WES/WGS and epigenetic data as well as RNA expressing data have made significant strides in understanding the biology of CLL, its molecular subtypes, epitypes and expression subtypes that significantly influence CLL outcome [18, 19].

The introduction of BTKis shifted the previous understanding of predictive treatment markers. Initial treatment with a BTKi in *TP53* aberrant patients can now achieve 70% PFS at five years, which is significantly better than the outcome of conventional immunotherapy [184]. Despite these improvements, *TP53* aberrations, particularly multi-hit mutations, remain negative prognostic factors for both treatment-naïve and relapsed or refractory CLL patients receiving BTKi therapy or venetoclax-based time-limited treatments [12, 35, 182].

The advent of targeted therapies has profoundly modified the prognostic implications, particularly concerning *TP53* aberrations and *IGHV* mutation status. While these markers retain prognostic value, their influence on treatment outcomes is now more contingent on the choice of therapy, accentuating the necessity for personalized treatment strategies.

The emergence of targeted therapeutic agents, including BTKi and BCL2i, has resulted in a paradigm shift in the management of CLL, even in patients harboring these adverse genetic prognostic indicators. These novel interventions have demonstrated efficacy in improving both PFS and OS while maintaining a tolerable side-effect profile. Despite these advancements, *TP53* aberrations continue to exert a deleterious influence on the prognosis of patients with CLL undergoing chemoimmunotherapy, underscoring the critical role of genetic markers in the stratification and selection of therapeutic strategies. Furthermore, mutations or deletions in *TP53* significantly influence the likelihood of disease relapse. Notably, CLL patients exhibiting mutations in *BTK* and/or *PLCγ2*, a phenotype that is more prevalent among individuals unresponsive to BTKi therapy, are three times more likely to possess *TP53* aberrations than their counterparts devoid of such mutations [35]. Additionally, in the context of the CLL14 phase 3 clinical trial, patients with *TP53* aberrations receiving treatment with venetoclax and obinutuzumab exhibited a markedly reduced PFS at five years relative to those without such genetic alterations [185]. The prognostic significance of multi-hit *TP53* aberrations, characterized by the co-occurrence of *TP53* mutations and del(17p), even at low variant allele frequencies (VAF), has been elucidated in recent studies. Bomben et al. (2023) demonstrated that the simultaneous presence of *TP53* deletions and mutations, irrespective of VAF, exerts a profound negative impact on the clinical outcomes of patients with CLL undergoing ibrutinib therapy [186]. This finding highlights the necessity of comprehensive *TP53* aberration screening prior to the initiation of ibrutinib treatment because only patients with concurrent *TP53* mutations and del(17p) exhibited significantly shorter OS and PFS than those without these genetic anomalies. The detrimental effect of these co-occurring aberrations persisted as a significant predictor of poor clinical outcomes in both univariate and multivariate analyses, along with the number of prior therapy lines and presence of anemia [186]. In agreement with these observations, Brieghel et al. [12] advocated the pre-treatment assessment of *TP53* aberrations in CLL patients, highlighting the association between *TP53* mutations and resistance to chemoimmunotherapy. They distinguished between single- and multi-hit *TP53* aberrations, with the former comprising a solitary *TP53* anomaly, and the latter involving multiple *TP53* aberrations. Despite being regarded as equivalent prognostic markers, patients with CLL with a single *TP53* abnormality undergoing monotherapy with ibrutinib generally exhibit superior PFS and OS compared to those with multiple *TP53* aberrations. This underscores the importance of comprehensive genetic testing, utilizing both FISH for del(17p) detection and deep next-generation sequencing for *TP53* mutation identification, to refine risk stratification and inform the development of novel therapeutic approaches for CLL patients with complex *TP53* aberrations [12].

The significance of the *IGHV* mutation status has also evolved in terms of its prognostic value with the emergence of targeted therapies. Unmutated *IGHV* is associated with worse PFS and OS following FCR and inferior PFS with fixed-duration therapy, including venetoclax and anti-CD20 monoclonal antibody. On the other hand, continuous administration of BTKi monotherapy does not seem to affect long-term outcomes, irrespective of *IGHV* mutation status [35]. This indicates that, while *IGHV* mutation status remains a critical prognostic marker, its predictive value varies depending on the treatment approach, highlighting the need for an in-depth understanding of the role of these markers in the context of targeted therapies [2].

In the era of chemoimmunotherapy, the significance of mutations in the *ATM* gene, *SF3B1* or *NOTCH1* was strongly emphasized as associated with outcomes; however, with the application of targeted therapies that do not target DNA, these mutations have become less significant, and mutations in *SF3B1* and *ATM* are being mentioned less by research. The advent of targeted therapies such as BTK inhibitors and venetoclax has significantly improved patient outcomes, and the effectiveness of these targeted therapies has shifted the focus from genetic profiling to clinical outcomes. Researchers and clinicians are interested in therapeutic responses and managing resistance to these treatments, leading to fewer publications specifically on this mutation. These therapies target specific pathways that are crucial for CLL cell survival and have proven effective across various genetic backgrounds, including those with *ATM*, *SF3B1*, and *NOTCH1* mutations [187]. On the other hand recent findings emphasize the critical role of *SF3B1* in instability in CLL. *SFB1* and *ATMP* mutations and chromosomal aberrations disrupt the normal formation of DNA:RNA hybrids, contributing to CIN and highlighting the potential for novel therapeutic strategies targeting R-loop dynamics. Furthermore, the interplay between *SF3B1*, *ATM*, and *TP53* mutations is essential for understanding the mechanisms driving genomic instability, which may inform patient stratification and treatment approaches in the future [108, 144].

Furthermore, despite significant advancements in therapies leading to improved overall and progression-free survival, some patients still experience unsatisfactory treatment effects and relapse. New therapies exert selection pressure on subclones, leading to their expansion, whereas other aberrations have gradually become more significant. When BTKi is administered, approximately one-third of patients experience relapse due to the emergence of treatment-resistant mutations, such as those in the *BTK* and *PLCγ2* genes [188].

In the near future, we anticipate significant shifts in predicting markers, particularly genetic markers, for CLL. Combined therapy with venetoclax and BTK inhibitors is likely to play a crucial role in this evolution. Thus, this therapy may alter the current landscape of marker significance. Because CLL is a highly heterogeneous disease, some patient groups still experience relapse after standard therapies and require personalized approaches. At this moment, we have a huge number of CLL markers, including cell phenotype, beta-2 microglobulin, CD38, ZAP70, and mentioned in this paper genetic markers [189]. Additionally, factors related to the CLL environment and nutrition also come into play. The microenvironment of the lymph nodes, spleen, and bone marrow provides crucial survival, proliferation, and drug resistance signals for CLL cells. Interactions between CLL cells and neighboring cells, such as stromal and nurse-like cells, enhance CLL cell survival and protect them from apoptosis. Interactions within the tumor microenvironment (TME), lead to significant metabolic changes in CLL cells, affecting their energy production and overall metabolism. These changes can influence the response of the cells to treatment. Furthermore, factors related to T-cell exhaustion and abnormal immune surveillance also affect CLL, contributing to its high heterogeneity.

Further research into the efficacy and safety of new drugs is essential to develop targeted, personalized therapies for patients with CLL. One example for such approach is potential clinical utility of PARP inhibitors in *SF3B1*MUT cancer patients. Bland et al. (2023) study results [144] suggest a clinical advantage for PARPi in this subset of patients, and that PARP-trapping agents could offer a new treatment option for patients with *SF3B1*MUT cancers that are resistant to conventional therapies.

Observational analyses based on large populations treated with targeted therapy could provide insights into identifying genomic features that should be further assessed in clinical trials. It appears that the administration of new agents in CLL overcomes the adverse effects of genomic aberrations in some cases.

## Data Availability

No datasets were generated or analysed during the current study.

## Abbreviations

ADC: antibody-drug conjugates
AID: Activation-Induced Cytidine Deaminase
ALLIANCE: Alliance for Clinical Trials in Oncology
ATM: Ataxia Telangiectasia Mutated
BCAP: B-cell Adaptor for Phosphatidylinositol 3-Kinase
Bcl-2: B-cell Lymphoma-2
BCL-XL: B-cell Lymphoma-Extra Large
BCR: B-cell Receptor
BER: Base-Excision Repair
BiTEs: Bispecific T cell Engagers
BLNK: B-cell Linker Protein
BTK: Bruton's Tyrosine Kinase
BTKi: Bruton's Tyrosine Kinase Inhibitors
C481: Cysteine 481
CAR: Chimeric Antigen Receptor
CD19: Cluster of Differentiation 19
CD5: Cluster of Differentiation 5
CD20: Cluster of Differentiation 20
CD22: Cluster of Differentiation 22
CD38: Cluster of Differentiation 38
CD79a: Cluster of Differentiation 79a
CD79b: Cluster of Differentiation 79b
CDR1: Complementarity-Determining Region 1
CDR2: Complementarity-Determining Region 2
CDR3: Complementarity-Determining Region 3
CIN: Chromosomal Instability
CIT: Chemoimmunotherapy
CLL: Chronic Lymphocytic Leukemia
CNV: Copy Number Variations
CR: Complete Responses
DAG: Diacylglycerol
DBLC: Diffuse Large B-cell Lymphoma
DLEU: Deleted in Lymphocytic Leukemia
DLL1: Delta-Like Ligand 1
DLL4: Delta-Like Ligand 4
DNA: Deoxyribonucleic Acid
DPLBCL: Double-Hit Large B-cell Lymphoma
ECs: expression clusters
epiCMIT: epigenetically-determined Cumulative MIToses
ER: Endoplasmic Reticulum
ERK: Extracellular Signal-Regulated Kinase
FCR: Fludarabine, Cyclophosphamide, and Rituximab
FDA: Food and Drug Administration
FISH: Fluorescence In Situ Hybridization
FL: Follicular Lymphoma
GC: germinal centre
GSs: genomic subgroups
GSI: Gamma-Secretase Inhibitor
HCDR3: Heavy Chain Complementarity-Determining Region 3
HP-CLL: high-programmed CLL
i-CLL: intermediate CLL
IGH: Immunoglobulin Heavy Chain
IGHV: Immunoglobulin Heavy Chain Variable
IKZF3: Ikaros Family Zinc Finger 3
IP3: Inositol 1,4,5-Trisphosphate
irAEs: immune-mediated adverse events
ITAM: Immunoreceptor Tyrosine-Based Activation Motif
ITIM: Immunoreceptor Tyrosine-Based Inhibitory Motif
iwCLL: International Workshop on Chronic Lymphocytic Leukemia
LP-CLL: low-programmed CLL
LYN: Lck/Yes-Related Novel Protein Tyrosine Kinase
MAPK: Mitogen-Activated Protein Kinase
MCL: Mantle Cell Lymphoma
m-CLL: memory B cells like CLL
M-CLL: mutated *IGHV* CLL
MOMP: Mitochondrial Outer Membrane Permeabilization
MZL: Marginal Zone Lymphoma
n-CLL: naïve B cells like CLL
NFKBIE: Nuclear Factor Kappa-Light-Chain-Enhancer of Activated B Cells Inhibitor, Epsilon
NGS: Next-Generation Sequencing
NICD: NOTCH Intracellular Domain
NOTCH1: Neurogenic Locus Notch Homolog Protein 1
ORR: Objective Response Rate
OS: Overall Survival
PAMPs: Pathogen-Associated Molecular Patterns
PCYC: Pharmacyclics
PKKC: Protein Kinase C
PFS: Progression-Free Survival
PI3K: Phosphoinositide 3-Kinase
PIP3: Phosphatidylinositol (3,4,5)-Triphosphate
PLCγ2: Phospholipase C Gamma 2
ROR1: Receptor Tyrosine Kinase-Like Orphan Receptor 1
RS: Richter Syndrome
SF3B1: Splicing Factor 3B Subunit 1
SHIP-1: SH2 Domain-Containing Inositol Phosphatase 1
SHP-1: Protein tyrosine phosphatase 1
SHM: Somatic Hypermutation
SYK: Spleen Tyrosine Kinase
TAK1: Transforming Growth Factor-Beta-Activated Kinase 1
TFH: T Follicular Helper Cells
TLR: Toll-Like Receptor
TME: Tumor Microenvironment
TTFT: Time to First Treatment
TTNT: Time to Next Treatment
U-CLL: Unmutated *IGHV *Chronic Lymphocytic Leukemia
VAF: Variant Allele Frequencies
WES: Whole-Exome Sequencing
WGS: Whole-Genome Sequencing
XPO1: Exportin 1
ZAP70: Zeta-Chain-Associated Protein Kinase 70
